# Identification of drought-responsive microRNAs and their targets in *Ammopiptanthus mongolicus* by using high-throughput sequencing

**DOI:** 10.1038/srep34601

**Published:** 2016-10-04

**Authors:** Fei Gao, Ning Wang, Huayun Li, Jisheng Liu, Chenxi Fu, Zihua Xiao, Chunxiang Wei, Xiaoduo Lu, Jinchao Feng, Yijun Zhou

**Affiliations:** 1 College of Life and Environmental Sciences, Minzu University of China, Beijing 100081, China; 2 College of Life Sciences, Qilu Normal University, Jinan 250013, China

## Abstract

MicroRNAs (miRNAs) regulate target gene expression to modulate plant growth, development, and biotic and abiotic stress response at the post-transcriptional level. *Ammopiptanthus mongolicus*, an ecologically important desert plant, is increasingly used as a model for studying stress tolerance in plants. The miRNA-mediated gene regulatory network might remarkably contribute to the high stress tolerance of *A*. *mongolicus*. However, a genome-wide identification of miRNAs and their targets is still lacking in *A*. *mongolicus*. In this study, 170 conserved and 156 non-conserved miRNAs were identified in *A*. *mongolicus*. We experimentally identified 298 miRNA-target pairs from the degradome data. Quantitative real-time polymerase chain reaction analyses identified 28 drought-responsive miRNAs in leaves and 15 in roots. Some characteristics of the miRNA-mediated regulatory network were found in *A*. *mongolicus*. Multiple miRNAs, including 2 newly identified non-conserved miRNAs, miR-P11 and miR-P14, generated from the precursors of miR169, were found to be involved in drought stress response. Further, miR2118 and miR858 participated in drought stress response by up-regulating OZF1 gene and certain MYB genes that were involved in the regulation of flavonol biosynthesis in *A*. *mongolicus*. The findings of this study might provide new insights for understanding the functions of miRNA in stress response in plants.

MicroRNAs (miRNAs) are endogenous 20–22 nucleotide (nt) small RNAs that play important roles in regulating gene expression in animals, plants, and fungi. The first miRNA, lin-4, was identified in a metazoan called *Caenorhabditis elegans*^1^, and the first plant miRNAs were reported in *Arabidopsis thaliana*^2^. MiRNAs play regulatory roles at the post-transcriptional level by directly degrading target messenger RNAs (mRNAs) or repressing translation^3^. Plant miRNA genes are first transcribed into primary miRNAs (pri-miRNAs) by RNA polymerase II. Next, pri-miRNAs are cleaved to hairpin-like miRNA precursor (pre-miRNAs), and the stems of the hairpin are cleaved into miRNA::miRNA* duplex by the RNase III enzyme DICER LIKE1 (DCL1). The mature miRNAs (guide strands) are incorporated into an RNA-induced silencing complex (RISC), and the RISC targets specific mRNAs and down-regulates the expression of target mRNAs^4^. Plant miRNAs have been shown to participate in the regulation of a wide range of biological processes such as growth, development^5^, metabolism^6^, hormone regulation, and responses to various abiotic and biotic stresses^7^.

Drought is one of the most important abiotic stresses that severely reduce plant growth and crop productivity. Elucidation of the molecular mechanism underlying drought stress response and adaptation might advance the effort to improve drought tolerance of crops, trees, and flowers via genetic engineering. Recently, numerous studies have shown that plant miRNAs are involved in the regulation of drought response^8^,^9^, and many miRNAs were shown to be responsive to drought stress in plants such as Arabidopsis^10^, rice^11^, maize^12^, barley^13^, wheat, cotton^14^, and barrel medic^15^. These results not only remarkably advanced our understanding on the biological roles of miRNAs in stress response, but also revealed that miRNAs participated in abiotic stress responses in a species-, miRNA-, and tissue-specific manner^8^. For example, miR156, miR159, miR162, miR167, and miR319 were up-regulated by drought stress in Arabidopsis^10^, barley^13^, and maize^12^, but down-regulated in cotton^14^. The species-dependent responses of miRNAs to environmental stress resulted in part from the functional diversification of miRNAs among plant species, and they were likely associated with the evolutionary dynamics of miRNA genes^16^. This diversity highlighted the importance of conducting studies on miRNAs in diverse non-model plant species, especially the extremophile plant species, in order to broaden the understanding of miRNA functions in stress response.

*Ammopiptanthus mongolicus*, a relic of the Tertiary period, can grow in harsh environments under extreme drought and freezing conditions in desert regions; it has been used as a model in abiotic tolerance research in trees^17^. A batch of putative stress tolerance-related genes were isolated and characterized^18^,^19^,^20^, and transcriptome analyses of drought and cold stress responses were conducted using next-generation sequencing technology in *A*. *mongolicus*^17^,^21^,^22^,^23^. These studies advanced our knowledge regarding the molecular genetic mechanisms underlying the drought stress response in *A*. *mongolicus* and provided numerous transcriptome sequences.

Given the important role of miRNAs in plant stress response, as revealed by many previous studies, we speculated that miRNA-mediated gene regulatory network might remarkably contribute to the high stress tolerance of *A*. *mongolicus*. However, a genome-wide identification of miRNAs and their targets is still lacking in *A*. *mongolicus*. Further, we aimed to determine how *A*. *mongolicus* miRNAs responded to drought stress and whether *A*. *mongolicus* has evolved novel miRNAs to facilitate survival in an arid environment. To address these questions, we performed high-throughput sequencing and quantitative reverse transcription polymerase chain reaction (qRT-PCR) to identify the conserved, non-conserved, and drought-responsive miRNAs, as well as their targets in *A*. *mongolicus* leaves and roots. In all, 326 miRNAs, including 170 conserved and 156 non-conserved, were identified. A total of 316 miRNA-target pairs were identified using degradome sequencing and bioinformatics. Many drought-responsive miRNAs were identified using stem loop quantitative reverse transcription PCR (SL-qRT-PCR) analyses. Several characteristics of *A*. *mongolicus* miRNAs and the responses of these miRNAs to drought stress were further discussed. The results of this study might provide new insights for understanding the miRNA-mediated regulatory network associated with drought stress in *A*. *mongolicus*.

## Results and Discussion

### Genome survey sequencing and assembly and transcriptome sequence assembly of *A*. *mongolicus*

Considering that genome sequence information for *A*. *mongolicus* is yet lacking, we performed genome survey sequencing of *A*. *mongolicus* by using high-throughput sequencing technology. In total, 118 Gb of sequence data were generated, which corresponded to over 150× coverage of the estimated *A*. *mongolicus* genome^23^. After the data were assembled with SOAPdenovo (http://soap.genomics.org.cn/)^24^, we obtained 518 M assembled genome survey sequences (GSSs), covering about 63.17% of the estimated genome size, with an N50 of more than 2000 nt and average size of 1630.5 nt for the contigs. The shortest sequence was 200 nt, and the longest was up to 76,285 nt. The dataset included 322,164 sequences longer than 200 nt; among them, 184,821 sequences were longer than 500 nt. Considering that 95.9% stem-loop sequences in miRBase are shorter than 200 nt and only 0.1% of them are longer than 500 nt^25^, the assembled GSSs were a good reference dataset for miRNA identification in *A*. *mongolicus*.

Establishing a high coverage transcriptome dataset is a requisite for the identification of miRNAs and miRNA target genes in non-model plants. For this, all available *A*. *mongolicus* nucleotide sequences (Supplementary Table S1) were collected and assembled using Trinity^26^, CLC (http://www.clcbio.com), and Cap3 softwares^27^. The final transcriptome dataset was called “Unigene.” The basic parameters and length distributions of the Unigene are listed in Supplementary Table S2. This transcriptome dataset served as the reference sequences for miRNA identification and target prediction.

### Overview of *A*. *mongolicus* small RNA populations

To identify miRNAs in *A*. *mongolicus*, we generated four small RNA libraries by using RNAs isolated from drought–stressed roots (DT_R) and unstressed roots (CK_R) and those isolated from drought–stressed leaves (DT_L) and unstressed leaves (CK_L). These small RNA libraries were then sequenced using the high-throughput Illumina sequencing platform. In total, 12,954,184; 12,084,271; 11,823,128; and 11,050,444 raw sequence reads were obtained, respectively. After adaptors, low-quality sequences, and sequences smaller than 18 nt were removed, 4.0 M, 8.5 M, 4.9 M, and 9.5 M clean reads with sizes ranging from 18 to 25 nt were collected, respectively (Supplementary Table S3). The clean data of small RNA libraries from drought-stress leaves and roots were significantly less than those of the control groups, since a substantial portion of small RNAs were excluded after adaptor and length filter (Supplementary Table S3). We speculated that drought stress treatment could have been responsible for the reduction in the quantity of clean data by affecting the small RNA distribution or library construction process. Nonetheless, over 4 million clean reads from drought-stressed leaf and root samples were eligible for further analysis, because raw read numbers greater than 4 million were found to contribute very little to the identification of novel miRNAs^28^.

**Figure 1:**
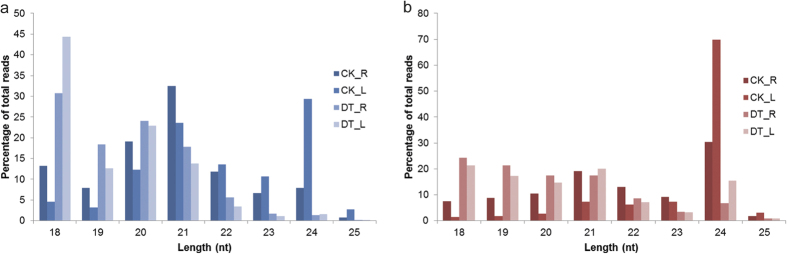
The length distribution of sequencing reads from the four *A*. *mongolicus* small RNA libraries. **a**) Size distribution of total clean reads. (**b**) Size distribution of unique small RNA sequences.

The majority (89.54% in CK_L library and 78.04% in CK_R) of clean reads ranged from 20 to 24 nt in length in the unstressed leaves and roots, whereas the percentage decreased significantly in drought-stressed tissues, reducing to 42.86% in DT_L and 50.61% in DT_R (Fig. 1a). The percentage of clean reads ranging from 18 to 20 nt in length in drought-stressed tissues increased from 7.76% in CK_L and 21.20% in CK_R to 56.99% in DT_L and 49.17% in DT_R.

In unstressed leaves, 24 (29.32%) and 21 (27.28%) nt small RNAs were the two major size groups (Fig. 1a) whereas, in unstressed roots, the two most abundant small RNAs were the 21 (32.47%) and 20 (19.08%) nt ones. After drought treatment, the two most abundant small RNAs switched to 18 and 20 nt ones in both leaves and roots (Fig. 1a). The alterations in small RNA size distribution pattern might result from the decrease in the percentage of unique sequences ranging from 23 to 24 nt and the increase in that of unique sequences ranging from 18 to 20 nt (Fig. 1b).

### Identification of conserved miRNAs and the corresponding stem-loop precursors in *A*. *mongolicus*

A total of 145 stem-loop precursors were identified; of which, at least one conserved mature miRNA sequence, derived from either 5′ or 3′ arms, was identified (Supplementary Table S4). Seven of them generated two miRNAs, and thus these 145 stem-loop precursors produced a total of 152 pairs of miRNA and miRNA*. These miRNA and miRNA* pairs produced 180 unique potential 5p and 3p miRNA sequences, including 170 conserved miRNA sequences and 10 less-conserved or non-conserved miRNA sequences. Almost all the stem-loop precursors showed extensive similarity to the miRNA precursors of green plants in the miRBase (Supplementary Table S4), except the stem-loop precursors of Amo-miR8175-1 and Amo-miR5139-1. These results showed that, as expected, the most conserved miRNAs are generated from conserved stem-loop precursors.

To identify more miRNAs and their stem-loop precursors, we used both transcriptome sequences and assembled GSSs for miRNA identification. Of the 145 identified stem-loop precursors, 83.45% (121/145) were identified from GSSs, whereas only 22.76% (33/145) were predicted from the transcriptome sequences (Supplementary Table S4). This result indicated that, compared to transcriptome sequences, GSSs are a better choice as reference sequence for miRNA identification in plant species without genome sequence information.

Ideally, for miRNA annotation, reads mapped to both miR and miR* sequences should be found. Although most of the identified conserved miRNAs were supported by the presence of both 5p and 3p arms of the hairpin precursors, miR* sequences of 36 identified miRNAs were not identified yet, including 19 5p arm miRNAs and 17 3p arm miRNAs. The validity of these conserved miRNAs was supported by the conservation of both their stem-loop precursors and mature sequences^29^.

The identified conserved miRNAs were grouped into 35 miRNA gene families (Table 1), according to the alignment results of their stem-loop precursors and mature sequences to the corresponding datasets in the miRBase database. The largest miRNA family is MIR159, with up to 19 members, followed by MIR156 (17 members), MIR166 (16 members), MIR171_2 (13 members), and MIR169 (8 members). Sixteen miRNA families, such as MIR1507, MIR1509, MIR1511, MIR1515, MIR403, and MIR858, had only one identified member.

Of the identified miRNA families, 24 showed marked conservation among multiple plant species, whereas the remaining 10 exhibited less conservation. For instance, MIR156, MIR166, MIR168, MIR169, MIR319, MIR394, MIR395, and MIR396 were identified from more than 30 monocots and dicots, whereas MIR1507 and MIR1509 were found only in legume plants such as *Glycine max*, *Medicago truncatula*^16^, and *Lotus japonicus* and were thus regarded as legume-specific miRNAs. The other miRNA families were only reported in few plant species. For example, MIR4415 was only identified from *G*. *max*; MIR5037 and MIR5559, from *G*. *max* and *M*. *truncatula*^16^; and MIR1515, from *G*. *max* and *Citrus sinensis*^30^. The identification of legume-specific and less-conserved miRNAs confirmed the efficacy of our miRNA identification workflow.

**Figure 2:**
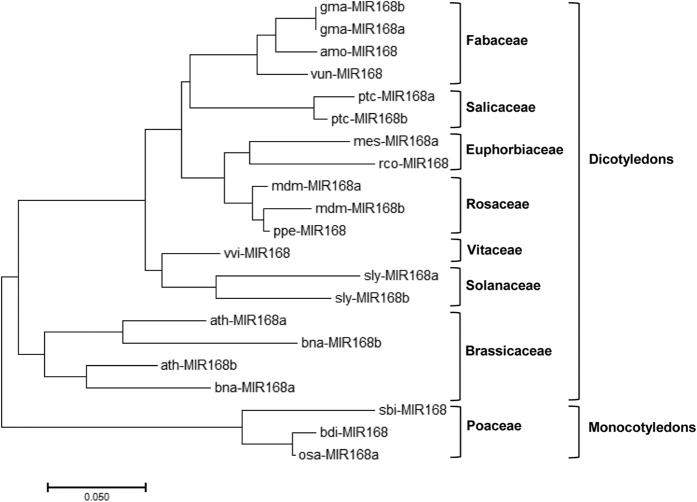
Phylogenetic tree of ame-MIR168 and its homologs. The evolutionary history was inferred using the Neighbor-Joining method. The optimal tree with the sum of branch length = 1.23819201 is shown. The tree is drawn to scale, with branch lengths in the same units as those of the evolutionary distances used to infer the phylogenetic tree. The analysis involved 21 nucleotide sequences. All positions containing gaps and missing data were eliminated. There were a total of 75 positions in the final dataset. amo –*Ammopiptanthus mongolicus*, ath – *Arabidopsis thaliana*, bdi – *Brachypodium distachyon*, bna – *Brassica napus*, gma – *Glycine max*, mdm – *Malus domestica*, mes –*Manihot esculenta*, osa –*Oryza sativa*, ppe – *Prunus persica*, ptc – *Populus trichocarpa*, rco – *Ricinus communis*, sbi – *Sorghum bicolor*, sly – *Solanum lycopersicum*, vun – *Vigna unguiculata*, vvi – *Vitis vinifera*.

To reveal the evolutionary history of the *A*. *mongolicus* miRNAs, we selected ame-MIR168, a conserved miRNA gene identified in the present study, for multiple sequence alignment and phylogenetic analyses. Multiple sequence alignment of ame-MIR168 and its homologs showed that, although the precursor length varied remarkably, two conserved regions were present among these precursors (Supplementary Fig. S1). One conserved region covered the guide strand of miR168 and the other one overlapped the region of the putative star strand of miR168. The phylogenetic tree of ame-MIR168 and its homologs revealed that the evolutionary distribution of miR168 family largely corroborated the currently accepted tree of life for angiosperm species, and the MIR168 precursors from legume family were clustered in the same clade in the phylogenetic tree (Fig. 2).

We analyzed the expression level of miRNA families based on the quantity of reads in the pooled small RNA library. The most abundant miRNA family was MIR166, which was sequenced more than 2 million times, followed by MIR159 and MIR156, which were sequenced over 0.6 million and 60,000 times, respectively. The fourth and the fifth abundant miRNA families were MIR1511 and MIR2118, with read numbers of over 35,000 and 19,000, respectively. In contrast, MIR1515, MIR5037, MIR4415, MIR399, and MIR397 exhibited low expression levels, with read numbers of less than 100. Notably, the expression level of MIR169 family, which is one of the most abundantly expressed miRNA families in plants, was relatively low in *A*. *mongolicus* (read number, <400).

Numerous plant miRNAs have been shown to be clustered and are known to generally contain several copies of the same conserved miRNA family^31^. In the present study, we found that scaffold190249 encoded three MIR2111 family members; scaffold190249, two MIR2111 family members; scaffold104500, three MIR166 family numbers; and scaffold40403, two MIR160 family members.

### Identification of non-conserved miRNAs in *A*. *mongolicus*

In total, 156 potent less-conserved or non-conserved miRNAs and the corresponding 123 stem-loop precursors were identified (Supplementary Tables S5, S6 and S7). According to the most recent criteria for distinguishing the high and low confidence miRNA annotations^32^, only 66 of them can be annotated as high-confidence miRNAs (putative miRNA, P1-P66; Supplementary Tables S5 and S6). These putative miRNAs were classified into two groups according to their stem-loop precursors. The first category included the 27 putative miRNAs derived from the stem-loop precursors that produced multiple miRNAs (Supplementary Table S5). The second category comprised the 39 predicted miRNAs generated from stem-loop precursors that produced only one miRNA (Supplementary Table S6). The read number of the star strand of the remaining 90 predicted non-conserved miRNAs, which were excised from 84 stem-loop precursors, was considerably low (less than ten or even zero), and they were classified as miRNA candidates (Supplementary Table S7, C1–C90). This result indicated that, when the sequencing depth is increased, more non-conserved miRNAs might be identified.

All the newly predicted non-conserved miRNAs have not been reported in other plant species; hence, most of these miRNAs can be deemed as species-specific miRNAs. However, some of the newly predicted non-conserved miRNAs might be lineage-specific miRNAs, and their homologs in other plant species were not detected owing to low expression level. To identify potential lineage-specific miRNAs, we conducted a BLASTN search of the newly predicted non-conserved miRNAs against the genome sequence data from 8 randomly selected legume plants. Our homology search revealed that almost 40% (62/156) predicted non-conserved miRNAs could be mapped to the genome sequences from at least one of the 8 legume plants with a cutoff of similarity percentage of 85%. By folding the flanking sequences of the hit sequences, we identified putative homologs of 13 non-conserved miRNAs (miR-P38, miR-P39, miR-P40, miR-P41, miR-P49, miR-P50, miR-P61, miR-P62, miR-C5, miR-C6, miR-C46, miR-C49, and miR-C50) from the genome sequences of these legume species (Supplementary Table S8). These miRNAs were not identified from the genome sequences of Arabidopsis, rice, and poplar. This result suggested that numerous lineage-specific miRNAs might have been spuriously identified as species-specific miRNAs in previous studies. Our data also validated the efficacy of the comparative genomics strategy used for the identification of novel miRNAs.

The sequencing frequencies of the majority of non-conserved miRNAs were very low, which was consistent with the hypothesis that non-conserved miRNAs are usually expressed at a lower level than conserved miRNAs^33^. Most of the identified non-conserved miRNAs (142/156, 91.03%) were sequenced less than 100 times (Supplementary Tables S5, S6 and S7). However, four miRNAs (miR-P29, miR-P30, miR-P31, and miR-C86) were found to have read numbers of more than 1,000. Among all the predicted non-conserved miRNAs, miR-P29 was the most abundant, with read number of more than 6,000 (Supplementary Table S6). These highly expressed non-conserved miRNAs might have developed under environmental pressure, and thus might be related to the biological characteristics of *A*. *mongolicus*.

### Target identification of miRNAs in *A*. *mongolicus*

To identify experimentally the target genes of the identified miRNAs in *A*. *mongolicus*, we performed degradome sequencing by using the pooled RNA sample from both leaves and roots. High-throughput sequencing produced 20,716,605 sequences (≥15 nt) representing the 5′ ends of uncapped, poly-adenylated RNAs (Table 2). The total number of signatures matching the transcriptome sequences was 11,987,867 (57.48%), and the number of distinct sequences matching the transcriptome sequences was 4,046,628.

We used CleaveLand pipeline^34^ to identify sliced miRNA targets in the *A*. *mongolicus* transcriptome. The target transcripts were divided into four categories (degradome category: 0–3) based on their abundance. In all, 132 pairs of miRNAs (132/298, 44.30%) and their targets belonged to category I, which accounted for the most abundant sequence reads at the cleavage site. Further 22, 154, and 1 miRNA-target pairs belonged to categories II, III, and IV, respectively. In addition, 223 category V (degradome category: 4) miRNA-target pairs were identified with a relatively lower confidence level, since the degradome read number of these miRNA-target pairs was only one. These transcripts are deemed to be the candidate target genes of the corresponding miRNAs.

A total of 298 miRNA-target pairs, related to 74 miRNAs and 183 transcripts, were identified with a high confidence level (Supplementary Tables S9 and S10). Among the 298 miRNA target genes, 258 were targeted by 54 conserved miRNAs and 40 were regulated by 20 non-conserved ones. This result showed that, compared with the conserved miRNAs, the evolutionarily new non-conserved miRNAs targeted fewer genes.

Of the 35 identified conserved miRNA families, MIR172, MIR 396, MIR 156, MIR 1509, and MIR 858 targeted multiple genes, whereas no targets were discovered for miRNA families MIR1511, MIR168, MIR171_2, MIR2111, MIR393, MIR395, MIR398, MIR403, MIR482, MIR5037, MIR5559, and MIR862_2. The expression level of the target genes of some miRNAs might have been too low to be identified in the present study.

Increasing evidence suggests that mature sequences derived from both arms of the hairpin might be biologically functional^35^; hence, we checked the identified targets of the conserved miRNAs to determine whether any transcripts were targeted by star strands of miRNAs. As expected, we found targets of 7 star strands, i.e., miR156-2, miR156-3, miR156-16, miR156-17, miR156-18, and miR156-19, and the identified targets of these star strands included transcripts encoding ubiquitin carboxyl-terminal hydrolase isozyme L5, prostaglandin E synthase 2-like, COP9 signalosome complex subunit 2, KAT8 regulatory NSL complex subunit 3-like, and TIMELESS-interacting protein-like, respectively.

The identified targets of the conserved miRNAs included many previously identified targets, such as the MYB genes for miR159 and miR319, SQUAMOSA promoter binding protein-like (SPL) genes as the targets of miR156, the endoribonuclease Dicer as the target of miR162 and miR1515, auxin response factor (ARF) as the target of miR167, and laccase as the target of miR397. New targets were also identified, including a molecular chaperone HtpG for miR396, serine/threonine-protein phosphatase 7 for miR1509, and calcium-transporting ATPase 10 as the target of miR5225.

Forty transcripts were identified as targets of 20 non-conserved miRNAs. Of these 40 transcripts, 5 were classed as category 0; 34, category 2; and 1, category 3. Notably, 12.50% and 48.06% of the targets of the non-conserved and conserved miRNAs, respectively, belonged to category 0. The low percentage of high-confidence targets of the non-conserved miRNAs might be attributed to the lower abundance of the non-conserved *A*. *mongolicus* miRNAs.

Among the identified targets of *A*. *mongolicus* non-conserved miRNAs, 29 were successfully annotated in UniProtKB/Swiss-Prot database. These target genes were involved in various predicted biological functions. Three identified category 0 targets of the non-conserved miRNAs were the mRNA of calcineurin B-like protein (CBL; targeted by miR-C59). CBLs acts as calcium sensors containing 3 EF-hand motifs and are involved in the signaling pathway during growth, development, and abiotic stress responses. The CBL calcium sensor SOS3 and the CIPK-type kinase SOS2 have been shown to be critical components of a calcium-regulated signaling pathway that mediates salt stress signaling and adaptation^36^. Thus, miR-C59 might be involved in responses to environmental stresses by targeting CBLs in *A*. *mongolicus*. Some transcription factor genes were also targeted by the non-conserved miRNAs (Supplementary Table S10). The most abundant non-conserved miRNA in *A*. *mongolicus* identified in the present study, miR-P29, targeted a homolog of AtBZIP60 transcription factor gene. AtBZIP60 has been shown to mediate unfolded protein response and to play critical roles in plant immunity and abiotic stress responses^37^. The high expression level of miR-P29 indicated its important role in drought response, although its biological function in *A*. *mongolicus* needs to be further investigated.

**Figure 3:**
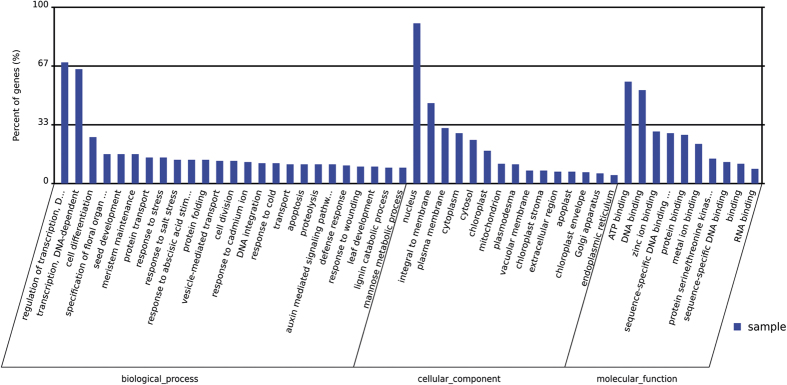
Gene Ontology functional classification of the miRNA targets identified in *A*. *mongolicus*. The x-axis represents the diverse biological functions of the targets according to three GO categories (biological process, cellular component and molecular function). The y-axis represents the percentage of the target genes.

**Figure 4:**
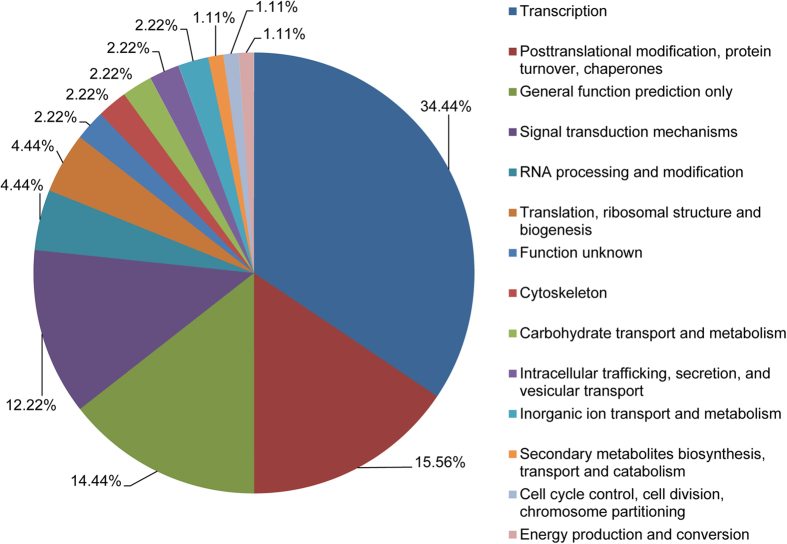


To classify the functions of the target genes of the identified miRNAs, we performed Gene Ontology (GO) functional classification analysis^38^. Based on sequence homology, all the target genes were categorized into 50 functional groups (Fig. 3). In the three main categories (Biological process (BP), Cellular component (CC), and Molecular function (MF)) of the GO classification, 25, 15, and 10 functional groups, respectively, were included (Fig. 3). Among these groups, the terms regulation of transcription, DNA-dependent (GO:0006355) and transcription, DNA-dependent (GO:0006351), nucleus (GO:0005634) and integral to membrane (GO:0016021), and ATP binding (GO:0005524) and DNA binding (GO:0003677) were the top 2 abundant terms in each of the three main categories, respectively. We also conducted KOG classification analysis to reveal the biological functions of the miRNA targets in *A*. *mongolicus*^39^ (Fig. 4). The results showed that the targets were involved in various functional categories, including transcription, posttranslational modification, signal transduction, RNA processing, translation, inorganic ion transport, secondary metabolites metabolism, and cell cycle control.

The functional analysis of the identified miRNA targets might provide valuable clues for understanding the biological characteristics of *A*. *mongolicus*. For example, the target genes classified in the GO terms “meristem maintenance” (GO:0010073) and “auxin mediated signaling pathway” (GO:0009734) regulate plant growth; hence, they might be related to the growth pattern of *A*. *mongolicus*. These genes are targeted by miR156, miR167, miR172, and miR-C71. The targets categorized in the GO terms “response to stress (GO:0006950),” “response to salt stress (GO:0009651),” and “response to cold (GO:0009409)” might be involved in responses to environmental stresses and should contribute to the extremely high tolerance to drought and cold stress in *A*. *mongolicus*. These genes were negatively regulated by a class of miRNAs, including miR319, miR396, miR408, miR4415, miR858, and miR-P52.

### Differential expression of miRNAs in *A*. *mongolicus* roots and leaves under drought stress

To investigate the expression pattern of miRNAs under drought stress in *A*. *mongolicus*, we separately compared the normalized expression values of miRNAs of untreated samples (CK_L and CK_R) and those of drought-stress-treated samples (DT_L and DT_R). According to the criteria described in “Methods,” 56 miRNAs were determined as the potential differentially expressed miRNAs in *A*. *mongolicus* roots or leaves under drought stress (Supplementary Table S11).

**Figure 5:**
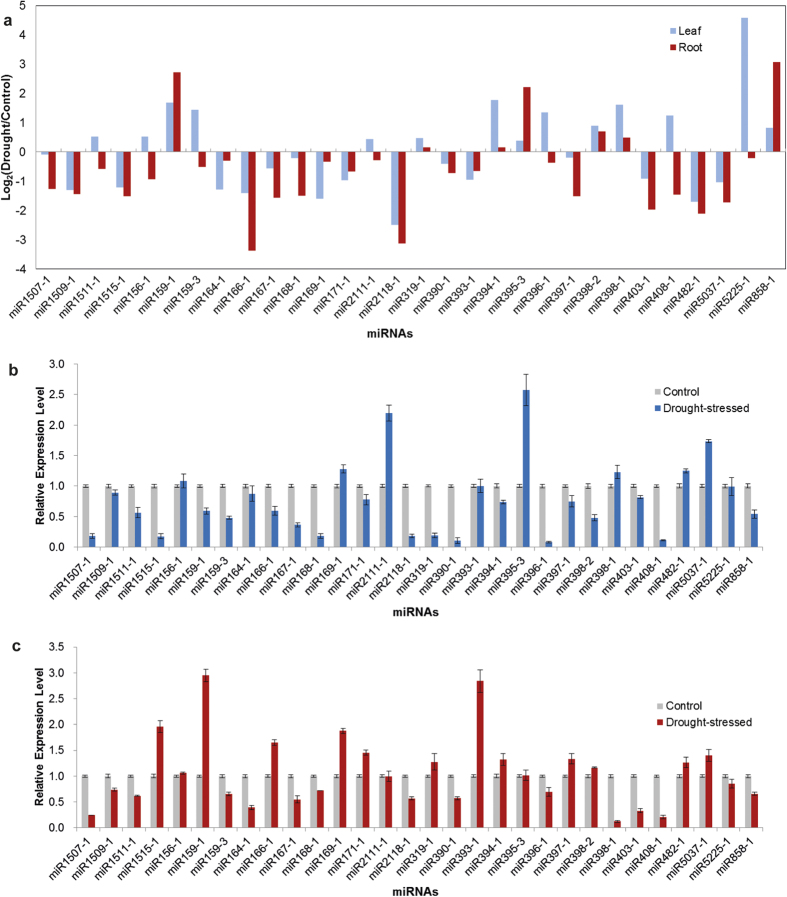
The expression patterns of conserved miRNAs under drought stress in *A*. *mongolicus* leaves and roots. **a**) Differential expression of conserved miRNAs in response to drought stress by comparing the normalized expression of miRNAs in small RNA libraries from the control and the drought-stressed groups. The relative expression level of miRNAs determined by SL-qRT-qPCR in response to drought stress in *A*. *mongolicus* leaves (**b**) and roots (**c**). *A*. *mongolicus* U6 was used as an internal control. Error bars represent ±SD from three independent experiments.

Thirty conserved miRNAs were selected, and SL-qRT-PCR analyses were conducted to validate the alterations in miRNA expression level under drought conditions revealed by the high-throughput sequencing results (Fig. 5). The miRNAs used for SL-qRT-PCR analyses included not only the 22 differently expressed miRNAs under drought stress identified by high-throughput sequencing, but also the several miRNAs whose homologs in other plant species have been reported to be responsive to abiotic stress, such as miR159-2, miR2111-1, miR319-1, miR390, and miR393^8^.

**Figure 6:**
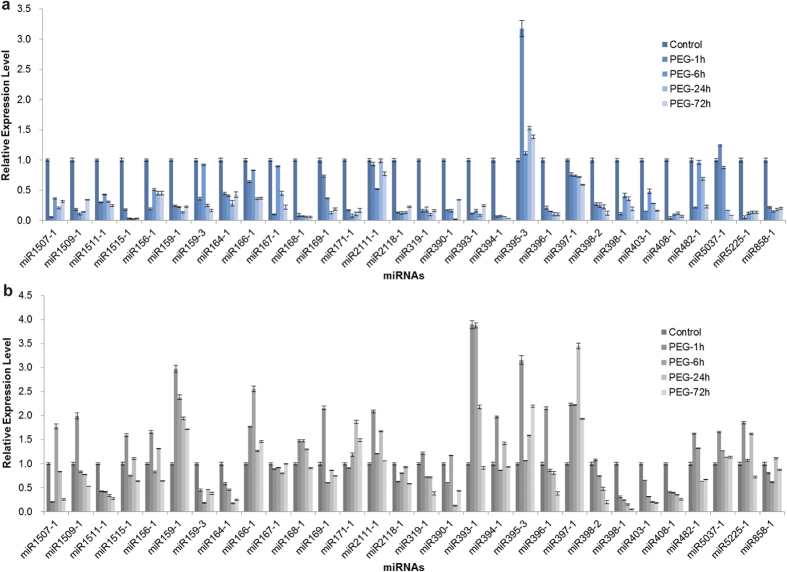
The expression patterns of conserved miRNAs under PEG-6000-induced drought stress in *A*. *mongolicus* leaves (**a**) and roots (**b**). *A*. *mongolicus* U6 was used as an internal control. Error bars represent ±SD from three independent experiments.

To further reveal the dynamic expression pattern of drought-responsive miRNAs during drought stress, in addition to the drought stress imposed by ceasing watering for two weeks, we also determined the expression levels of miRNAs under PEG-6000-simulated drought stress for 1, 6, 24, and 72 h. The expression patterns of small RNA sequencing and SL-qRT-PCR results of the potential drought-responsive miRNAs were not consistent (Figs 5 and 6). For some miRNAs, the results of SL-qRT-PCR analysis were considerably different from those of the high-throughput sequencing. Similar findings have been obtained by some recent studies^40^,^41^. Considering that no biological replicates were set in small RNA sequencing in the present study, as well as the two steps of RNA adaptor ligation and one step of PCR amplification during sample preparation for deep sequencing, which might have introduced bias in the final data, we mainly relied on the results of SL-qRT-PCR to identify the differently expressed miRNAs under drought stress in *A*. *mongolicus*. The results of SL-qRT-PCR analyses also indicated that the responses of miRNAs to drought stress were highly dynamic and could vary with the treatment regimen.

The drought-responsive miRNAs were ultimately determined based on SL-qRT-PCR results. In leaves, a total of 28 conserved miRNAs belonging to 25 families were found to be regulated under drought stress, including 26 down-regulated, i.e., miR1507-1, miR1509-1, miR1511-1, miR1515-1, miR156-1, miR159-1, miR159-3, miR164-1, miR166-1, miR167-1, miR168-1, miR169-1, miR171-1, miR2118-1, miR319-1, miR390-1, miR393-1, miR394-1, miR396-1, miR398-1, miR398-2, miR403-1, miR408-1, miR482, miR5225-1, and miR858-1, and 2 up-regulated, i.e., miR395-3 and miR2111-1. In contrast, in roots, only 15 conserved miRNAs belonging to 13 families were found to be regulated under drought stress, including 11 down-regulated, i.e., miR1507-1, miR1511-1, miR159-3, miR164-1, miR167-1, miR168-1, miR390-1, miR398-1, miR398-2, miR403-1, and miR408-1, and 4 up-regulated, i.e., miR159-1, miR393-1, miR395-3, and miR397-1.

Only 10 non-conserved miRNAs (identified with high confidence) with transcripts per million (TPM) values of more than 3 were identified, and the expression levels of the majority of these miRNAs decreased under drought stress as revealed by sequencing read count analyses (Supplementary Fig. S2 and Table S11). Three of these miRNAs (miR-P11, miR-P14, and miR-P16) were selected to be further analyzed by using SL-qRT-PCR; miR-P11 and miR-P14 were shown to be down-regulated significantly in leaves and miR-P14 was shown to be down-regulated in roots under drought stress (Supplementary Fig. S2).

Most of the studies on differential miRNA expression analyses focused on the comparison of miRNA abundance by using entire plants, and such a strategy cannot reveal the difference in the expression patterns between different tissues or organs. In the present study, several drought-responsive miRNAs were found to show different drought-induced change patterns between leaves and roots, for example, miR159-1 and miR393-1 were down-regulated in leaves, but up-regulated in roots under drought stress. Similar tissue-specific expression patterns were also observed previously in other plant species, such as *Phoenix dactylifera*^42^. These results suggested that the biological roles of miRNAs in stress response varied with individual organs or tissues.

The majority of the drought-responsive miRNAs identified in the present study have been reported previously as being differential expressed in other plant species, indicating that many similarities exist between *A*. *mongolicus* and other higher plants with respect to miRNA-mediated gene regulatory network in response to drought stress, as described and discussed in several recent reviews^8^,^43^. Further, some characteristics of miRNAs and miRNA-mediated regulatory network were observed under drought stress in *A*. *mongolicus*.

### Multiple miRNAs generated from the stem-loop precursors of miR169 were involved in drought responses

We identified 14 miRNA stem-loop precursors generating small RNAs from distinct regions of the same stem-loop precursors. Table 3 lists all these miRNA precursors, the corresponding miRNAs, and the miRNA-like RNAs identified from the four small RNA libraries. The alignment of sequencing reads to part of these precursors is shown in Supplementary Fig. S3. Among these precursors, MiR_658, MiR_595, MiR_522, MiR_703, MiR_1101, MiR_374, and MiR_1350 probably generated two miRNA-5p/3p pairs, whereas three or four miRNA-5p/3p pairs were excised from MiR_213, MiR_457, MiR_1092, MiR_865, and MiR_1267 hairpin-like sequences. This result showed that at least 9.7% (14 of 145) of the conserved *Ammopiptanthus* miRNA precursors produced this type of small RNA. This ratio is comparable to that in Arabidopsis (9.1%)^41^. All precursors generated at least one conserved miRNA. Almost all these precursors generated at least one non-conserved miRNA except MiR_522.

Generation of multiple miRNAs from the same stem-loop precursors has been reported previously in several plants such as *A*. *thaliana*, *Oryza sativa*, *M*. *truncatula*, *Populus trichocarpa*, and *Panax ginseng*^41^. Generation of multiple miRNAs from the same precursors has previously been observed in several miRNA families, including MIR156, MIR159, MIR160, MIR168, MIR169, MIR171, MIR319, MIR390, MIR394, MIR408, and MIR482^41^; more than one miRNA derived from the stem-loop precursor of MIR2118 was identified for the first time in our study, to our knowledge.

Unlike the non-conserved miRNAs generated from the same stem-loop precursors, conserved miRNAs are expected to be expressed at a higher level^41^. As expected, the conserved (cognate) miRNAs showed a higher read number in ten of the twelve precursors. However, two of these non-conserved miRNAs were more abundant than their cognate miRNAs, and both of them were derived from MIR169 family precursors (Table 3). Among them, miR-P11 “uucggcuuucuuccucucaug” has 340 reads, which is 19-fold more than the cognate one “aagccaaggaugacuugccgg.”

**Figure 7:**
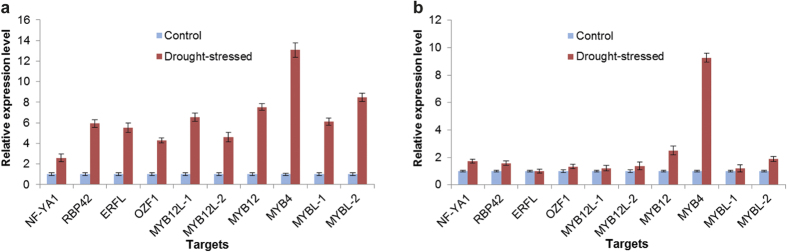
The drought stress-induced expression patterns of ten miRNA targets in *A*. *mongolicus* leaves (**a**) and roots (**b**). *A*. *mongolicus eIF1* was used as an internal control. Error bars represent ±SD from three independent experiments.

MiR169 is one of the largest conserved miRNA families in the plant kingdom. Nuclear factor YA (NF-YA) transcription factor genes, the target of miR169, have been confirmed to function in the positive modulation of plant drought stress tolerance^44^. Several studies have shown that miR169 was responsive to abiotic stresses such as drought, cold, and salinity in various plant species^8^,^43^. In *A*. *mongolicus* leaves, the expression level of miR169-1 was down-regulated by drought stress (Fig. 6a), and this change pattern is consistent with that of *M*. *truncatula*^15^. As expected, the expression level of the nuclear transcription factor Y subunit A-1-like protein encoding gene (NF-YA1), one of the targets of miR169-1 identified by the degradome sequencing, was up-regulated significantly in the leaves (Fig. 7). The miR169-1 might participate in the response to drought stress in *A*. *mongolicus* by enhancing the expression of NF-YA1 transcription factor gene.

Moreover, we found that the expression level of miR169 was surprisingly low in *A*. *mongolicus* leaves and roots (Table 3). We speculated that the low constitutive expression might lead to high constitutive expression of the NF-YA genes, contributing to drought stress tolerance of *A*. *mongolicus*. Such a “stress-ready” strategy in unstressed condition has been proposed in other extremophiles such as *Thellungiella salsuginea*^45^.

The two stem-loop precursors of miR169 also generated two non-conserved miRNAs with expression level higher than that of miR169 (Table 3). The high expression level of the newly evolved miRNAs suggests their potential biological roles in *A*. *mongolicus*; thus, the drought-induced expression patterns of these two miRNAs, i.e., miR-P11 and miR-P14, as well as the expression profiles of the corresponding targets, a RNA-binding protein 42 gene (*RB42*) and an ethylene-responsive transcription factor-like protein gene (*ERFL*), were further investigated using qRT-PCR analyses (Fig. 7 and Supplementary Fig. S2). RNA-binding proteins (RBPs) play regulatory roles in the post-transcriptional processes of gene expression in plants under various stress conditions, and a palm oil stress-induced *RBP42* was shown to interact with transcripts involved in various functions, particularly in transcription, translation, and stress responses, to contribute to stress tolerance^46^. *ERFL* encodes an AP2/ERF-type transcription factors that regulates important functions of plant growth and development as well as responses to environmental stimuli^47^. We found a clear negative correlation between the expression levels of the two miRNAs and their targets in *A*. *mongolicus* leaves, indicating that miR-P11 and miR-P14 might participate in drought stress response by activating the expression of *RB42* and *ERFL* genes.

### MiR858 mediated the drought-induced modulation of flavonol biosynthesis by up-regulating MYB transcription factors

In a previous transcriptome study, we found that genes involved in the biosynthesis of secondary metabolites, including flavone and flavonol, were overrepresented in drought stress-regulated genes in *A*. *mongolicus* leaves^17^. We speculated that some transcription factors regulating flavonol biosynthesis might be activated by drought stress. MYB transcription factors have been reported to play vital roles in flavonol biosynthesis; thus, we checked the identified miRNA-target pairs to detect the miRNA that might mediate the drought-induced modulation of flavonol biosynthesis by targeting MYB genes.

Four miRNA families have been shown to target MYB transcription factors, i.e., MIR159, MIR319, MIR828, and MIR858. MIR828 was not found in *A*. *mongolicus*, and our degradome data identified 18 MYB transcription factor genes targeted by the other three miRNA families. Most the identified MYB targets belonged to the R2R3-MYB class, which played a role in biological processes, including primary and secondary metabolism, cell fate and identity, developmental processes, and responses to biotic and abiotic stresses^48^. The qRT-PCR analyses of six randomly selected targets (*MYBL-1* and *MYBL-2* were the targets of miRNA members of family MIR159 and MIR319; *MYB12*, *MYBL-1*, *MYBL-2*, and *MYB4* were the targets of miR858) revealed that all these MYB targets were up-regulated by drought stress in the leaves, whereas only two of these MYB targets were down-regulated by drought stress in the roots (Fig. 7).

Among the 18 identified MYB targets, 5 were shared by miRNA members of family MIR159 and MIR319, whereas the other 13 MYB genes were targeted by miR858. These results indicated that, unlike miR159 and miR319, miR858 might play different roles by regulating certain MYB transcription factors. Of the 13 identified MYB targets of miR858, 11 were homologs of Arabidopsis *MYB11* (AT3G62610) or *MYB12* (AT2G47460), and ATMYB11 and ATMYB12 have been shown to function redundantly with ATMYB111 in activating the expression of enzyme genes committed to flavonol biosynthesis, such as chalcone synthase, flavanone 3-hydroxylase, and flavonol synthase^49^,^50^. Thus, miR858 probably mediates the modulation of flavonol biosynthesis by regulating certain MYB transcription factors in drought-stressed *A*. *mongolicus*. Conversely, miR159 and miR319 might mainly play roles in anther development and seed germination by targeting the homologs of Arabidopsis *MYB33* and *MYB65* in *A*. *mongolicus*, considering that the two MYB transcription factors were found to be involved in gibberellin signaling in anthers and germinating seeds^51^.

The drought-induced expression patterns provided additional data to understand the role of miR858 in drought response. The qRT-PCR analyses showed that miR858 was mainly expressed in leaves, and the expression level of miR858 reduced remarkably in leaves, but not in roots (Figs 5 and 6). Correspondingly, all the 4 MYB targets were up-regulated by drought stress in leaves, whereas only two of them were up-regulated by drought stress in roots (Fig. 7). Flavonoids are believed to have a protective role in plants exposed to drought condition, and drought stress-induced flavonoid accumulation has been observed in several plant species^52^. Thus, our findings suggested that miR858 mediated the modulation of flavonol biosynthesis by activating certain MYB transcription factor genes under drought stress in *A*. *mongolicus* leaves.

### MiR2118 participated in drought stress response by up-regulating an OZF1 gene

MIR2118 is a less-conserved miRNA family identified mainly in leguminous plants, and their function is thought to be to regulate the expression of the nucleotide-binding site leucine-rich repeat (NBS-LRR) disease resistance protein genes. We found that the expression level of Amo-miR2118 in both roots and leaves decreased significantly under drought stress (Figs 5 and 6). The 6 identified targets of this miRNA included 3 NBS-LRR domain-containing disease resistance protein gene homologs, an *OZF1* gene homolog, and 2 uncharacterized genes. The Arabidopsis OZF1 protein was localized in the plasma membrane and was required for the tolerance of Arabidopsis to oxidative stress^53^. We further checked the drought stress-induced alteration in the expression level of the three NBS-LRR domain-containing disease resistance protein gene homologs and *OZF1* homolog by using qRT-PCR to determine whether a clear negative correlation existed between the expression levels of Amo-miR2118 and its targets. As expected, the expression level of *OZF1* homolog increased significantly (Fig. 7).

MiR2118 was reported to be up-regulated by drought stress in *Phaseolus vulgaris*^54^, *Caragana intermedia*^55^, and *M*. *truncatula*^15^. In the present study, miR2118 was also responsive to drought stress, but with a considerably different regulation pattern. We proposed that, although more attention needs to be paid to the NBS-LRR domain-containing disease resistance protein gene targets of miR2118 in other plant species, the activation of the expression of *OZF1* by miR2118 might play a crucial role in drought response of *A*. *mongolicus*.

### miRNA-mediated gene regulatory network in response to drought stress in *A*. *mongolicus*

miRNAs are the negative regulators of genes. In general, if an miRNA is down-regulated, its targets are likely to be up-regulated post-transcriptionally. To reveal the negative correlation between the expression level of miRNAs and that of their targets, we analyzed the expression patterns of 16 targets of the drought-responsive miRNAs in drought-stressed *A*. *mongolicus* leaves and roots by using qRT-PCR (Fig. 7 and Supplementary Fig. S4). The results showed that, for the majority of the drought-responsive miRNAs, a clear negative correlation was found between the expression level of the miRNA and that of its target. For example, an ARF8 encoding gene (targets of miR167) was up-regulated in leaves under drought stress, which is in line with the down-regulation of miR167; two targets of miR396, encoding a GRF9 and an RD21a-like protein, respectively, showed up-regulation in drought stressed *A*. *mongolicus* leaves, which is opposite to that of miR396. However, a few exceptions were found, for example, the expression of plantacyanin, a predicted target of miR408, exhibited the same expression profile as that of miR408 in leaves and roots. We speculated that the expression of plantacyanin might be regulated by a more complicated mechanism, since three additional miRNAs—miR397, miR398 and miR857—were found to target the plantacyanin transcripts^56^.

*A*. *mongolicus* is a relic plant species with extremely high tolerance to drought and cold stress. The identified conserved, lineage-, and species- specific miRNAs in *A*. *mongolicus* were likely to be associated with the biological characteristics and stress tolerance of *A*. *mongolicus*. To illustrate the regulatory roles of drought-responsive miRNAs in *A*. *mongolicus* globally, we constructed an miRNA-mediated gene regulatory network based on miRNA-target relationship and the negative correlation between the expression level of the miRNAs and that of its targets revealed by the present study (Supplementary Fig. S5). Considering that most of drought-responsive miRNAs were identified in leaves, our model was primarily focused on the physiological adjustment regulated by miRNAs in drought-stressed *A*. *mongolicus* leaves. In this network, most of the targets are transcription factors, including SBP, MYB, NAC, TCP, ARF, ERF, HD-ZIP, and NF-YA. Many miRNA-target pairs in this network are involved in “development” and “defense and stress response,” highlighting the central roles of these physiological adjustments in drought response in *A*. *mongolicus*. This network contains some transcription factors that regulate disease response, such as NBS-LRR and ERF, indicating that cross-talk exists between miRNA regulatory pathways for biotic and drought stress responses in *A*. *mongolicus*.

*A*. *mongolicus* has a strong taproot and well-developed lateral roots, which enable the plant to absorb water and nutrients from the soil. Several miRNAs regulating root architecture via modulating auxin signaling were shown to be responsive to drought stress, and these miRNAs included miR167, miR164, and miR393^57^, suggesting their functions in stress signaling and response. Our study showed that the drought stress-induced up-regulation of miR167 led to the down-regulation of the expression of the target, an ARF8 transcription factor, which is a positive regulator of adventitious root formation^58^. In addition, the decrease of miR393 expression in roots under drought stress up-regulated TIR1, an auxin receptor, which might boost auxin signaling. NAC1 has been shown to transduce auxin signaling for lateral root emergence, and inhibition of the NAC1 pathway suppressed the formation of adventitious root tips^59^. We identified an *A*. *mongolicus* NAC1 homolog targeted by miR164 and observed concurrent up-regulation of miR164 and down-regulation of NAC1 homolog in *A*. *mongolicus* roots under drought stress, indicating drought stress promoted root development via the miR164-NAC1 pathway in *A*. *mongolicus*. Taken together, our findings support the important functions of some of the drought-responsive miRNAs involved in the development of the root system of *A*. *mongolicus*.

As sessile organisms, plants have a remarkable ability to alter their development in response to environmental stress, and this capacity is especially critical for plants found in the arid environment. The phenotypic and developmental plasticity in plants can be mediated by miRNAs such as miR156 and miR159/319. miR156 has been shown to regulate plant developmental timing and phase change by targeting SPL transcription factors, and overexpression of miR156 inhibits developmental transitions and delays flowering. Further, miR156 has been shown to be responsive to drought stress in several plant species such as Arabidopsis and rice, but the drought stress-induced expression patterns varied remarkably^8^,^9^. Our findings revealed that *A*. *mongolicus* exhibited considerable plasticity in growth and development under drought stress via the miR156-SPL pathway.

## Conclusion

In recent years, high-throughput sequencing technology has been used extensively to identify plant miRNAs and abiotic stress-responsive miRNAs, and these studies have greatly advanced the understanding of the biological roles of miRNAs in stress response. In this study, high-throughput sequencing technology and bioinformatics were used to obtain GSS and transcriptome sequences, and to conduct small RNA and degradome sequencing. We ultimately identified 170 conserved miRNAs and 156 non-conserved miRNAs, of which batches of lineage- and species-specific miRNAs were discovered. A total of 298 miRNA-target pairs were experimentally identified, including many new target miRNA pairs. Many drought-responsive miRNAs, including 28 conserved miRNA in leaves and 15 in roots, were identified by using SL-qRT-PCR. Most of the drought-responsive miRNAs identified in the present study have been reported to show differential expression in other plant species, indicating the existence of common mechanisms shared by different plant species regarding the miRNA mediated-regulatory network underlying drought stress response. Further, some characteristics of miRNAs and miRNA-mediated regulatory network under drought stress were observed in *A*. *mongolicus*. In particular, several newly evolved non-conserved miRNAs such as miR-P11, miR-P14, miR-P29, and miR-C59 were identified, and these miRNAs might participate in stress response by targeting stress-related genes in *A*. *mongolicus*. Our study might provide important data for better understanding the molecular mechanism underlying the extremely high tolerance of this relic desert plant.

## Methods

### Ethics statement

The seeds of *A*. *mongolicus* were collected and research activities were scientifically conducted under the permits issued by Zhongwei Forest Bureau. The experimental procedures were approved by the Ethics Committee for Plant Experiments of the Minzu University of China and the State Forestry Administration, China.

### Plant material and stress treatment

The seeds of *A*. *mongolicus* were collected from Zhongwei city, Ningxia autonomous district, China. Seeds were surface-sterilized by treatment with 70% ethanol for 1 min, followed by bleaching (10%) for 6 min and rinsing for five times with sterile deionized water. The sterilized seeds were planted in a 6-inch pot filled with a 3:1 (v/v) mixture of sterilized vermiculite and perlite. The seedlings were cultured in growth chambers and grown at 20–25 °C with 16 h of light per day. The seedlings were watered every four days with half-length Hoagland solution.

For drought treatment, 96 four-week-old seedlings were randomly divided into two groups. One group was non-irrigated for two weeks, and the relative water content (RWC) of leaves ranged from 55% to 66% at the end of the two weeks of treatment. The other group served as the control, in which half-length Hoagland solution was regularly provided to seedlings to maintain a RWC higher than 75%. For PEG-6000-induced short-term drought treatment, 150 four-week-old seedlings were randomly divided into five groups. The first group was harvested before treatment and served as the control (0 h), whereas the second (1 h), third (6 h), fourth (24 h), and fifth (72 h) groups were irrigated with 20% PEG-6000 for 1, 6, 24, and 72 h, respectively. Each control or treated sample was pooled from 5–8 seedlings. After stress treatment, the root and leaf tissues of control and stressed groups were collected, frozen in liquid nitrogen, and stored at −80 °C for RNA extraction.

### Genome DNA sequencing, assembly, and transcriptome sequence assembly

Genomic DNA of *A*. *mongolicus* was extracted from young seedlings by using the Plant Genomic DNA Kit (Tiangen biotech, Beijing, China) following manufacturer’s instructions. Paired-end libraries with 350- and 550-bp insertions were constructed following a standard protocol provided by Illumina. DNA sequencing was performed on the Illumina HiSeq 2500 platform. The *de novo* assembly of the *A*. *mongolicus* genome was performed using SOAPdenovo^24^. Raw reads were preprocessed to remove adaptors and filter out reads of low quality. The assembled reads were deposited in the Whole Genome Shotgun (WGS) database (http://www.ncbi.nlm.nih.gov/genbank/wgs); they are retrievable under the accession number JZLJ00000000.

For transcriptome sequence assembly, we collected all available *A*. *mongolicus* nucleotide sequences (before September 8, 2015), including NCBI EST sequences generated by Sanger sequencing and high-throughput sequencing reads from SRA database (Supplementary Table S1). Considering that the sequence length distributions of these nucleotide data were different, a two-step strategy was adopted in the assembly process. First, we assembled the nucleotide data generated by pair-end Illumina sequencing by using Trinity software^26^ and assembled the 454 sequences by using CLC software (http://www.clcbio.com). Next, the resultant assemblies and NCBI EST sequences were further assembled using Cap3 software^27^. All assemblers were run using default parameters.

### Small RNA library construction and sequencing

The *A*. *mongolicus* seedlings were subjected to drought stress by stopping watering, and the corresponding control seedlings were used for small RNA library construction. Total RNA was extracted using Trizol reagent (Invitrogen, CA, USA) from the leaves and roots of *A*. *mongolicus* seedlings following the manufacturer’s procedure. The quantity and purity of total RNA were analyzed using Bioanalyzer 2100 and RNA 6000 Nano LabChip Kit (Agilent, CA, USA) with a RIN value of >7.0. Approximately 1 μg of total RNA was used to prepare small RNA library according to protocol of TruSeq Small RNA Sample Prep Kits (Illumina, San Diego, USA). Subsequently, we performed single-end sequencing (36 bp) on an Illumina Hiseq2500 at the LC Sciences (Hangzhou, China) following the manufacturer’s instructions. Raw sequence reads were obtained using Illumina’s analysis software.

### Identification of conserved and non-conserved miRNAs

Raw reads obtained from the Solexa sequencer were processed by removing contaminant reads, including those with 5′ primer contaminants, without 3′ primer, with poly A, and having length less than 18 nt. The resulting “clean reads” were used for length distribution analysis. Further, rRNAs, tRNAs, snRNAs, and snoRNAs were removed from the clean read sequences via BLASTN search by using Rfam database (http://www.sanger.ac.uk/Software/Rfam/). The raw data of small RNA sequencing have been submitted to the NCBI GEO datasets under the accession number GSE66080.

The unique sequences left were mapped to the *A*. *mongolicus* GSS and transcriptome sequences, and the potential miRNAs were identified by folding the flanking genome sequence of unique small RNAs by using the ACGT101-miR program (version 4.2, LC Sciences). Reads that mapped more than 20 times were discarded. The other parameters were set based on the criteria for annotation of plant miRNAs^29^. All predicted stem-loop precursors were checked separately, and false positive results were removed manually.

Among all potential candidate miRNAs, those that show similarity (allow no more than 3 mismatches) to the sequence of known green plant miRNAs from miRBase 21.0 (http://www.mirbase.org) were classified as “conserved miRNA.” The remaining potential miRNA candidates were classified as “non-conserved miRNA.” If a non-conserved miRNA met the requirements for annotating as “high confidence miRNAs,” according to the most recent criteria for distinguishing the high confidence miRNA annotations^32^, the non-conserved miRNAs were classified as “Putative non-conserved miRNA,” and the remaining non-conserved miRNAs were classified as “Non-conserved miRNA candidates.”

### Multiple alignment and phylogenetic analysis

The stem-loop precursor of miR168 from *A*. *mongolicus* and its homologs downloaded from miRBase were used to conduct multiple sequence alignments by using ClustalW 2.0 with default alignment parameters^60^. The phylogenetic analyses were performed using MEGA 7 software^61^. The evolutionary distances were computed using the Kimura 2-parameter model^62^ and are presented as the number of base substitutions per site.

### Identification of miRNA homologs in other legume species

By using the predicted non-conserved miRNA sequences in *A*. *mongolicus* as queries, we conducted a BLASTN search against the genome sequence of 8 randomly selected legume species with the cutoff E value of 10^−1^. The hits with a percentage similarity of less than 85% (i.e., the number of nucleotide differences between the sequences was more than 3) were excluded. Next, we extracted hit sequences with 300 nt of the 5′ and 3′ flanking sequences from the responding genome. If the extracted sequence had a typical secondary structure of miRNAs and the putative miRNA was located at the stem of the hairpin, the sequence was regarded as a putative miRNA homolog of the predicted non-conserved miRNA in *A*. *mongolicus*.

The 8 legume species included *G*. *max*, *M*. *truncatula*, *P*. *vulgaris*, *Cajanus cajan*, *Cicer arietinum*, *L*. *japonicus*, *Nelumbo nucifera*, and *Vigna radiata*. The genome sequences for the former three species were downloaded from Phytozome version 11.0 (http://www.phytozome.net), and those for the remaining species were downloaded from GenBank (http://www.ncbi.nlm.nih.gov).

### Degradome library construction and sequencing

A degradome library was constructed from pooled RNA samples from both leaves and roots according to a previously described method^63^ with minor modification. Briefly, approximately 150 ng of poly (A) RNA was used as input RNA and annealed with biotinylated random primers. After RNA fragments were streptavidin captured using biotinylated random primers, the RNAs with monophosphate at 5 ′ end were ligated to a 5 ′ adaptor. Next, reverse transcription and PCR were performed. The resulting library was single-end sequenced using an Illumina Hiseq2500 platform at the LC-BIO (Hangzhou, China) following the vendor’s recommended protocol. CleaveLand 3.0^34^ was used for analyzing sequencing data. The raw data of degradome sequencing have been submitted to the NCBI GEO datasets under the accession number GSE66095.

### Differential expression analysis of miRNAs

The sequence reads of the four libraries were normalized to 1 million by the total number of clean small RNA reads in each sample. Only top abundant miRNAs in a miRNA family with a normalized read count of more than 1 TPM, or any miRNA with a normalized read count of more than 3 TPM were analyzed. The log2 ratio formula was as follows: log2 ratio = log2 (miRNA reads in drought stressed group/miRNA reads in control). The miRNAs with a log2 ratio of more than 1 or less than -1 were considered as potential differentially expressed miRNAs and some of them were selected for SL-qRT-PCR analyses. The drought-responsive miRNAs were ultimately determined based on SL-qRT-PCR results, and the miRNAs showing >2-fold increase or decrease in expression were considered as drought-responsive miRNAs.

### SL-qRT-PCR analysis

Total RNA (100 ng) was used to initiate the reverse transcription reaction. The quantitative reaction was performed on an MyiQ2 two-color real-time PCR detection system (Bio-Rad Laboratories, Hercules, USA) by using the GoTaq^®^ qPCR Master Mix (Promega, Madison, USA). Three biological replicates were used for each treatment and the control, and all reactions were run in triplicate for each sample. U6 small nuclear RNA was used as the internal control for SL-qRT-PCR. The relative expression levels of miRNAs were quantified using the 2^−ΔΔCt^ method^64^. Standard deviations were calculated from three biological replicates. The primers used for SL-qRT-PCR analysis are listed in Supplementary Table S12.

### qRT-PCR analysis

The qRT-PCR analyses were conducted according to a previously described protocol^17^. Three independent biological replicates for each sample and three technical replicates of each biological replicate were analyzed using qRT-PCR. The expression levels of selected targets were normalized against an internal reference gene, *AmeIF1* (GenBank accession no.: JN885965). The relative gene expression was calculated using the 2^−ΔΔCt^ method^64^. Standard deviations were calculated from three biological replicates. The primers used for qRT-PCR analyses of miRNA targets are listed in Supplementary Table S13.

## Additional Information

**How to cite this article**: Gao, F. *et al*. Identification of drought-responsive microRNAs and their targets in *Ammopiptanthus mongolicus* by using high-throughput sequencing. *Sci. Rep*. **6**, 34601; doi: 10.1038/srep34601 (2016).

## Supplementary Material

Supplementary Information

Supplementary Table S1

Supplementary Table S2

Supplementary Table S3

Supplementary Table S4

Supplementary Table S5

Supplementary Table S6

Supplementary Table S7

## Figures and Tables

**Table 1:** The identified miRNA families in *A*. *mongolicus* leaves and roots.

miRNA families	Member quantity
MIR159	19
MIR156	17
MIR166	16
MIR171_2	12
MIR169	8
MIR160, MIR395, and MIR396	7
MIR164, MIR172, and MIR2111	6
MIR167	5
MIR399	4
MIR393, MIR394, and MIR398	3
MIR390, MIR408, and MIR4414	2
MIR1507, MIR1509, MIR1511, MIR1515, MIR162, MIR168, MIR2118, MIR397, MIR403, MIR4415, MIR477, MIR482, MIR5037, MIR5225, MIR5559, MIR858, and MIR962_2	1

**Table 2:** Summary of the degradome library data.

	Number	Ratio/%
Raw reads	20,856,478	—
reads <15 nt after removing 3′ adaptor	139,873	0.67
Mappable reads	20,716,605	99.33
Unique raw reads	7,904,288	—
Unique reads <15 nt after removing 3 adaptor	60,991	0.77
Unique mappable reads	7,843,297	99.23
Transcript mapped reads	11,987,867	57.48
Unique transcript mapped reads	4,046,628	51.20
Number of covered transcript	68,333	52.74

**Table 3:** Multiple miRNAs originated from distinct regions of the same stem-loop precursors.

Precursor	miRNA-5p/3p pairs number	Precursor homolog	5p sequence	Homolog	No. of 5p reads	3p sequence	Homologs	No. of 3p reads
MiR_658	2	MIR156	ugacagaagagagugagcac	gma-miR156u	8468	ugcucacuucucuuucugucagg	mtr-miR156i-3p	297
			acgcuucuggugauuguaug		16	cauacaauugcgggugcgu		9
MiR_213	4	MIR159	agcuccuugaaguccaauug	gma-miR159a-5p	648	uuuggauugaagggagcucu	gma-miR159a-3p	528280
			ugcugcugugcuauggauccc	gma-miR159d	802	cuuccauaucuugggagc	pvu-miR159a.2	4
			aagcucuugcuagguugauug		63	auugccuacaguuagauccuua		10
			acaguucuacccauagua		6			
MiR_457	4	MIR159	agcuccuugaaguccaauug	gma-miR159a-5p	640	uuuggauugaagggagcucaa	gma-miR159a-3p	55931
			agcugcugagcuauggauccc	gma-miR159d	2041	cuugcauaucucgggagcuuc	pvu-miR159a.2	3255
			aggauauugcuggguugauug		55	auuaccuuuaguuuuauccuuc		17
			acaguucuacccaucauga		2	uuuuuugugguagaccugcgg		15
MiR_1350	2	gma-MIR169c	aagccaaggaugacuugccgg	gma-miR169s	17	ggcaagucauccuguggcua	mtr-miR169d-3p	15
			uguagagguugaaaguggaaug		8	uucggcuuucuuccucucaug		340
MiR_374	2	gma-MIR169f	cagccaaggaugacuugccgg	gma-miR169a	11	cggcaaguccgccuuggcuac		4
			ugaagaggcagagagugcagug		78	uugggcucucuucuucucgug		244
MiR_595	2	miR2118	ggauaugggaggaucggaaagc	gma-miR2118a-5p	4533	uuuccgauuccacccauuccua	mtr-miR2118	14952
			gagaagagcuuggggaaguuaug		180	uuauuuccuuucguuccucuc		260
MiR_1092	3	miR319	gagcuuccuucaguccacuc	mtr-miR319a-5p	52	uuggacugaagggagcuccc	gma-miR319a	
			agcugcugacucauucauuca	ata-miR319-5p	15	ugugaaugaugcgggagguaa	mtr-miR4414b	14
			gaugaguagaggguuugaauu		10			
MiR_865	3	miR319	gagcuuccuucaguccacuc	mtr-miR319a-5p	52	uuggacugaagggagcuccc	gma-miR319a	18135
			agcugcugacucauucauuca	ata-miR319-5p	15	ugugaaugaugcgggagcuaa	mtr-miR4414b	6
			uggauggguaaagggcuugaauu		2			
MiR_1267	3	miR319	agagcuuucuucaguccacuc	mtr-miR319a-5p	35	uuggacugaagggagcuccc	gma-miR319a	18162
			uagcugccgacucauucaucca	ppe-miR319b	15	agugaaugaugcgggagaca	mtr-miR4414b	33
			ugggugguaguaggauuuaau		7	uugaaucuuaagccuccuguac		3
MiR_278	3	miR319	agagagcuuucuucaguccacuc	mtr-miR319a-5p	35	uuggacugaagggagcuccc	gma-miR319a	18165
			uagcugccgacucauucaucca	ppe-miR319b	15	agugaaugaugcgggagacaagu	mtr-miR4414b	87
						ugaaucuuaagcuuccuguac		2
MiR_522	2	miR159				uuuugguuugaagggagcucc	gma-miR319p	26
			ugcugcuaguucauggauucc	bdi-miR159b-5p.3	2			
MiR_703	2	gma-MIR398d	gggucguccugagaucacaug	ata-miR398f-5p	5	uguguucucaggucgccccug	gma-miR398c	2728
						cuuguggucucguuugugcc		2
MiR_1101	2	gma-MIR408a	cugggaacaggcagagcaugg	ahy-miR408-5p	120	augcacugccucuucccuggc	gma-miR408a-3p	5258
						agaaaccuguuguggcuacacu		5
MiR_2882	2	mdm-MIR482a	ggaaugggcgguuugggaaaa	mdm-mir482a-5p	106	uucccaaagccgcccauuccga	mdm-mir482a-3p	819
			gagauuggagcuaucagaaguugug		48	ugauuucuggugguuccucc		47
