# Supplementary material for: Identification of drought-responsive microRNAs and their targets in Ammopiptanthus mongolicus by using high-throughput sequencing
Source: Sci Rep. 2016 Oct 4;6:34601. doi: 10.1038/srep34601 (PMC5048172; doi:10.1038/srep34601)
Supplement: Supplementary Information [file srep34601-s1.pdf]

Supplementary information

Identification of drought-responsive microRNAs and their targets in *Ammopiptanthus mongolicus* by using high-throughput sequencing

Fei Gao, Ning Wang, Huayun Li, Jisheng Liu, Chenxi Fu, Zihua Xiao, Chunxiang Wei, Xiaoduo Lu, Jinchao Feng, Yijun Zhou*

Supplementary Figures

Suppl. Fig. S1 Multiple alignment of amo-MIR168 stem-loop precursor and its homologous precursors. amo –*Ammopiptanthus mongolicus*, ath – *Arabidopsis thaliana*, bdi – *Brachypodium distachyon*, bna – *Brassica napus*, gma – *Glycine max*, mdm – *Malus domestica*, mes –*Manihot esculenta*, osa –*Oryza sativa*, ppe – *Prunus persica*, ptc – *Populus trichocarpa*, rco – *Ricinus communis*, sbi – *Sorghum bicolor*, sly – *Solanum lycopersicum*, vun – *Vigna unguiculata*, vvi – *Vitis vinifera*.

Suppl. Fig. S2 The expression patterns of non-conserved miRNAs under drought stress in *A. mongolicus* leaves and roots. (a) Differential expression of non-conserved miRNAs in response to drought stress by comparing the normalized expression of miRNAs in small RNA libraries from the control and the drought-stressed groups. The relative expression level of three randomly selected non-conserved miRNAs determined by SL-qRT-PCR in response to drought stress in *A. mongolicus* leaves (b) and roots (c). *A. mongolicus U6* was used as an internal control for SL-qRT-PCR. Error bars represent ±SD from three independent experiments.

Suppl. Fig. S3 The alignment of sequencing reads to part of precursors that generated multiple miRNA-5p/3p pairs. The horizontal lines represent mapped reads and the numbers above or below the horizontal lines represent the quantity of the corresponding reads. The most abundant miRNA generated from the stem-loop precursor is shown in red.

Suppl. Fig. S4 The drought stress-induced expression patterns of six miRNA targets in *A. mongolicus* leaves (a) and roots (b). Target 1, targeted by miR156-1, encodes a squamosa promoter-binding-like protein 12-like protein; target 2, targeted by miR164-1, encodes a NAC domain-containing protein; target 3, target of miR167-1, encodes a ARF8; target 4 and 5, targeted by miR396-1, encodes a GRF9 and a Cysteine proteinase RD21a-like protein, respectively; target 6, targeted by miR408-1, encoding a plantacyanin, one of blue copper proteins. *A. mongolicus eIF1* was used as an internal control. Error bars represent ±SD from three independent experiments.

Suppl. Fig. S5 The miRNA-mediated gene regulatory network in response to drought stress in *A. mongolicus* leaves. The green arrowed line indicates that the negative correlation between the expression level of the miRNA and that of its target has been confirmed experimentally.

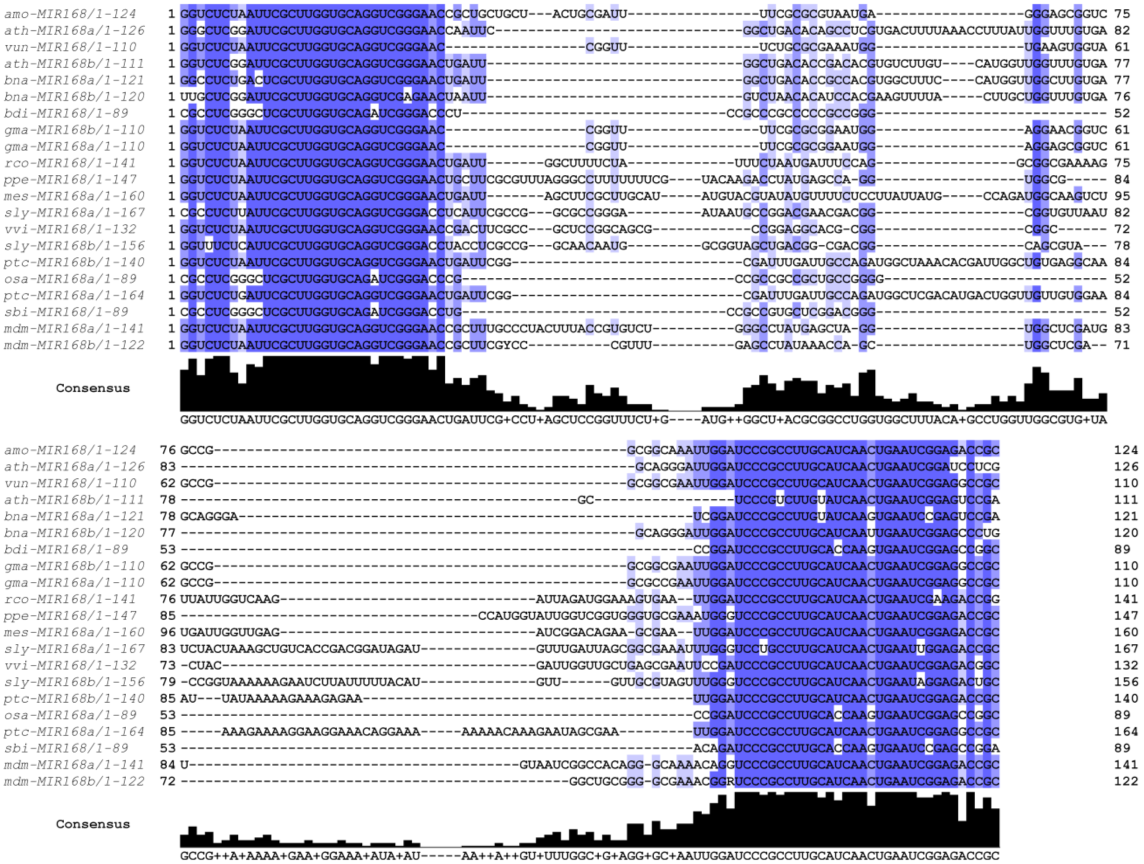

Suppl. Fig. S1 Multiple alignment of amo-MIR168 stem-loop precursor and its homologous precursors. amo –*Ammopiptanthus mongolicus*, ath – *Arabidopsis thaliana*, bdi – *Brachypodium distachyon*, bna – *Brassica napus*, gma – *Glycine max*, mdm – *Malus domestica*, mes –*Manihot esculenta*, osa –*Oryza sativa*, ppe – *Prunus persica*, ptc – *Populus trichocarpa*, rco – *Ricinus communis*, sbi – *Sorghum bicolor*, sly – *Solanum lycopersicum*, vun – *Vigna unguiculata*, vvi – *Vitis vinifera*.

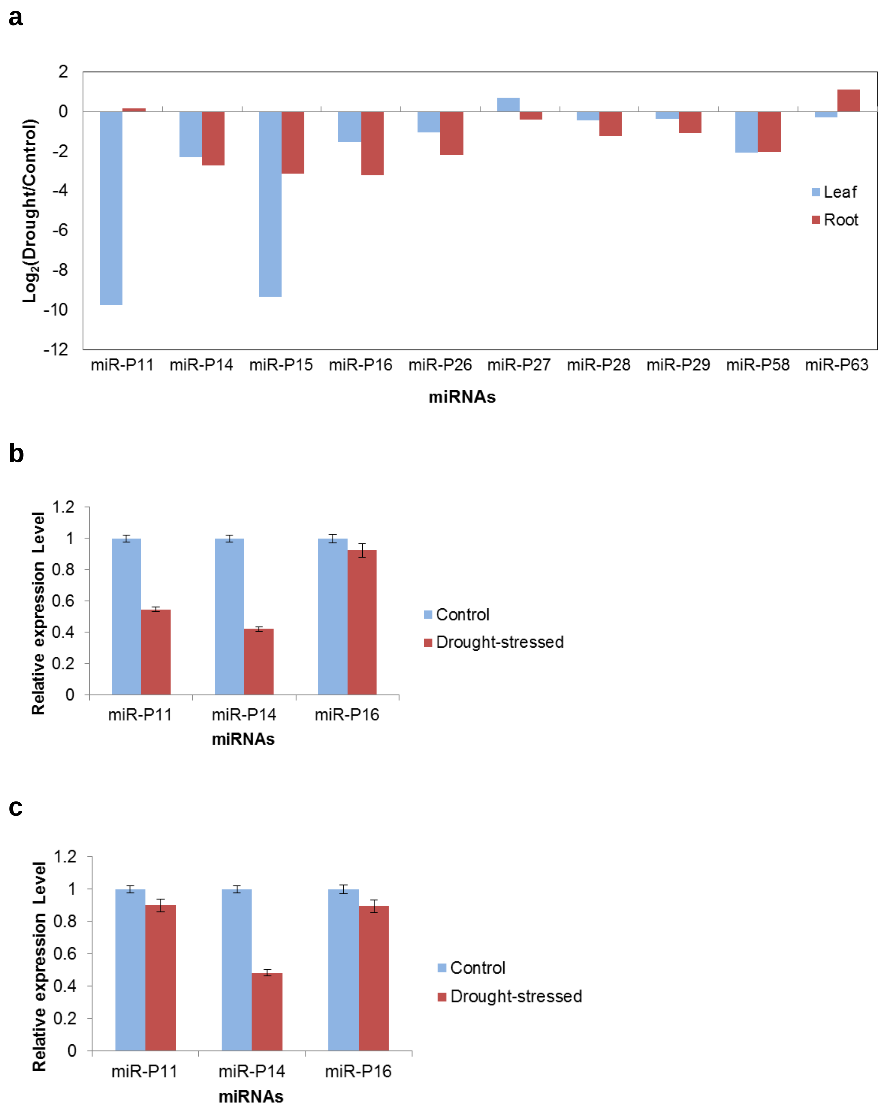

Suppl. Fig. S2 The expression patterns of non-conserved miRNAs under drought stress in *A. mongolicus* leaves and roots. (a) Differential expression of non-conserved miRNAs in response to drought stress by comparing the normalized expression of miRNAs in small RNA libraries from the control and the drought-stressed groups. The relative expression level of three randomly selected non-conserved miRNAs determined by SL-qRT-PCR in response to drought stress in *A. mongolicus* leaves (b) and roots (c). *A. mongolicus U6* was used as an internal control for SL-qRT-PCR. Error bars represent ±SD from three independent experiments.

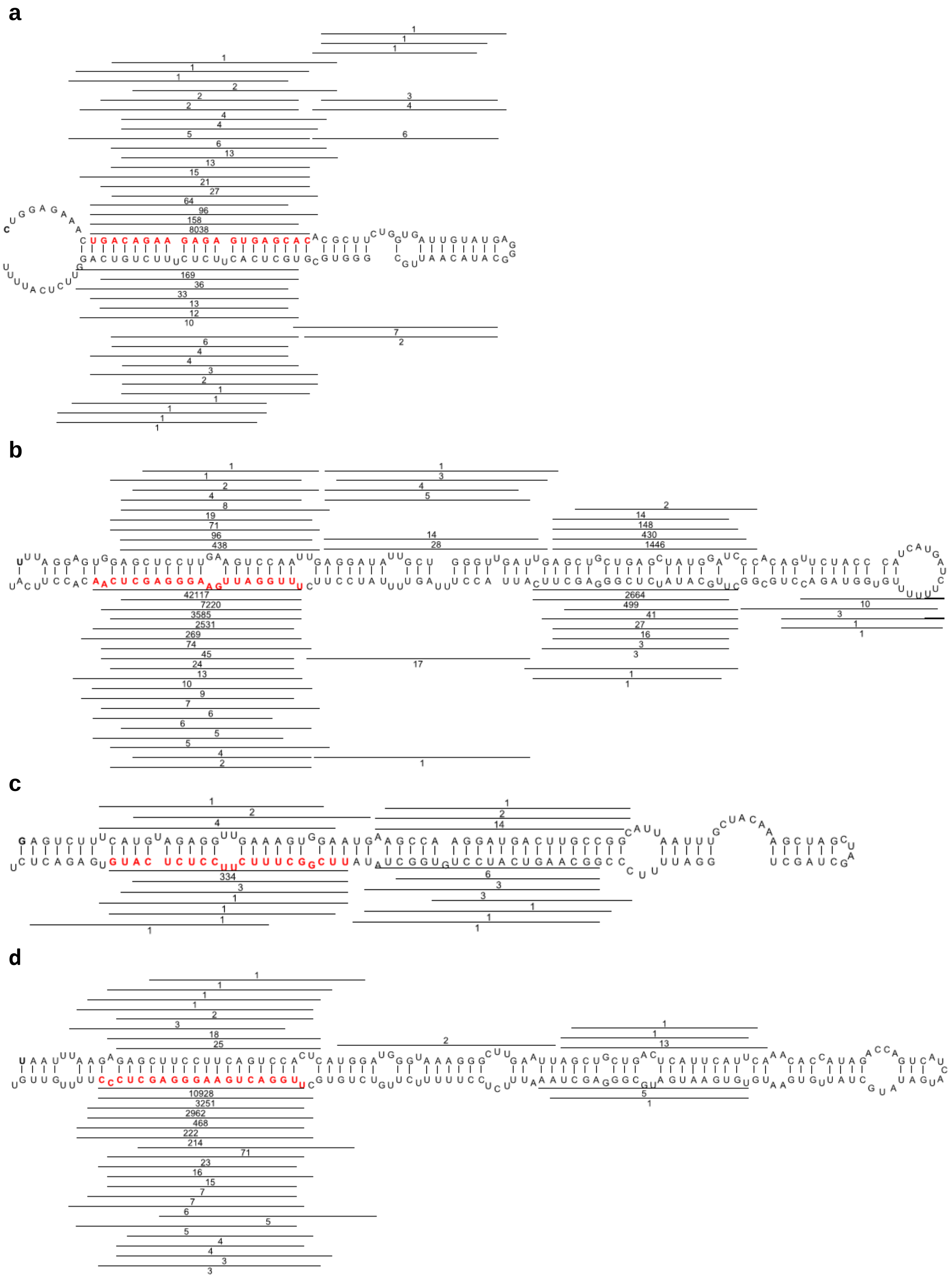

Suppl. Fig. S3 The alignment of sequencing reads to some of precursors that generated multiple miRNA-5p/3p pairs. (a) MiR658. (b) MiR457. (c) MiR1350. (d) MiR865. The horizontal lines represent mapped reads and the numbers above or below the horizontal lines represent the quantity of the corresponding reads. The most abundant miRNA generated from the stem-loop precursor is shown in red.

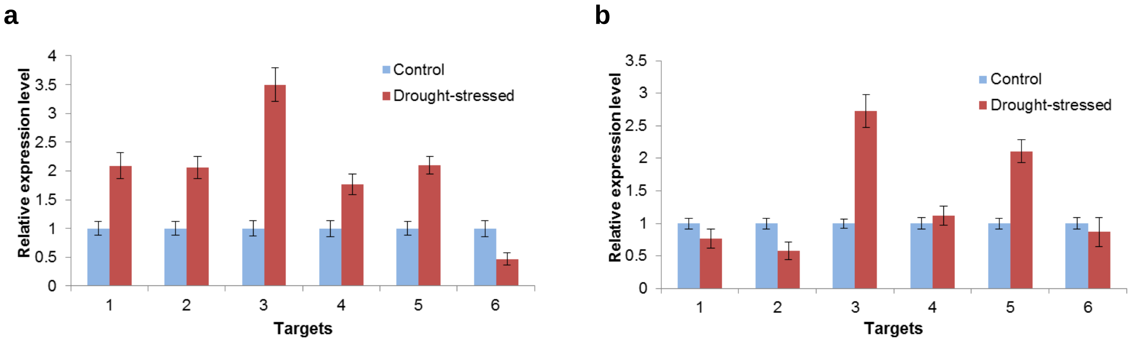

Suppl. Fig. S4 The drought stress-induced expression patterns of six miRNA targets in *A. mongolicus* leaves (a) and roots (b). Target 1, regulated by miR156-1, encodes a squamosa promoter-binding-like protein 12-like; target 2, targeted by miR164-1, encodes a NAC domain-containing protein; target 3, target of miR167-1, encodes a ARF8; target 4 and 5, targeted by miR396-1, encodes a GRF9 and a cysteine proteinase RD21a-like protein, respectively; target 6, targeted by miR408-1, encoding a plantacyanin, one of blue copper proteins. *A. mongolicus eIF1* was used as an internal control. Error bars represent ±SD from three independent experiments.

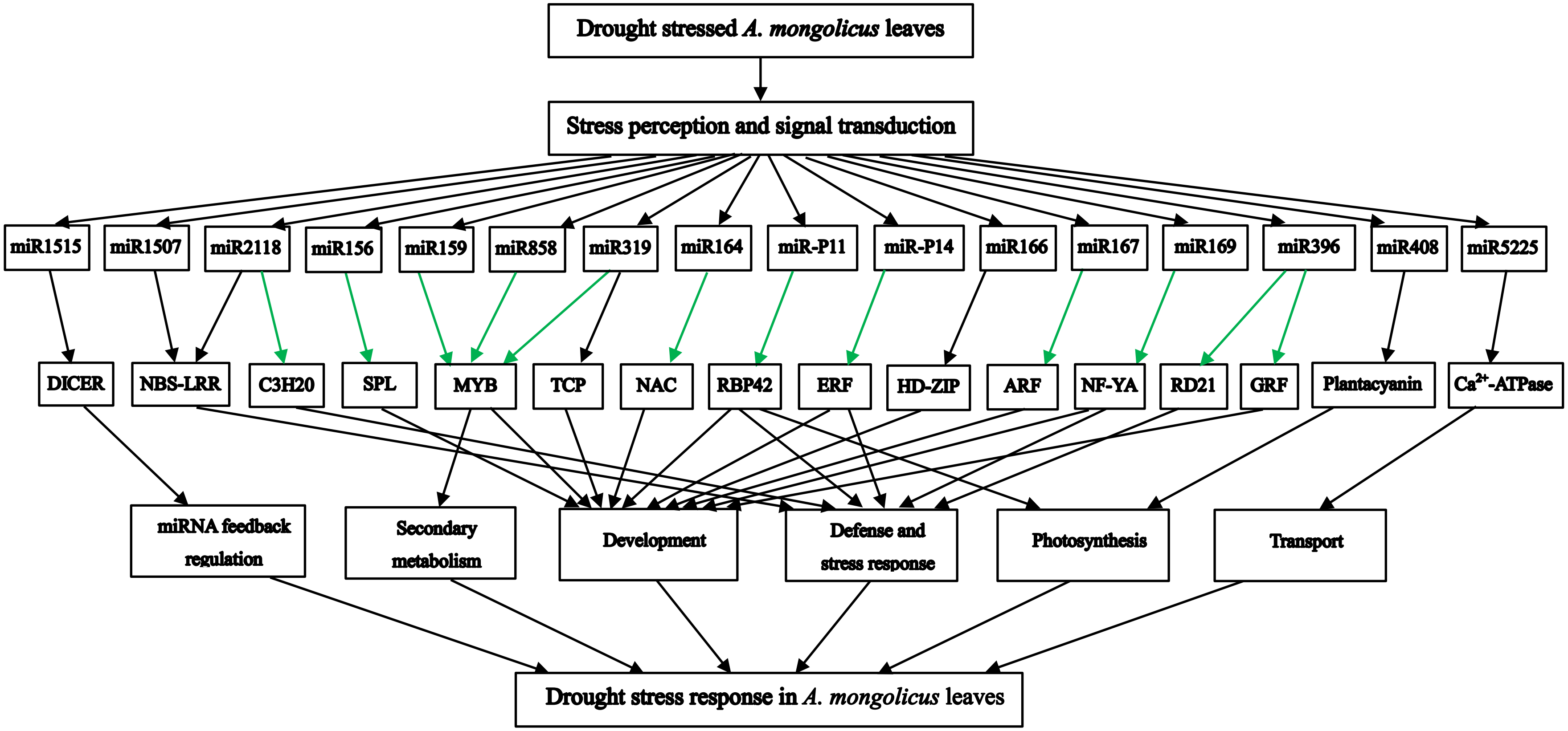

Suppl. Fig. S5 The miRNA-mediated gene regulatory network in response to drought stress in *A. mongolicus* leaves. The green arrowed line indicates that the negative correlation between the expression level of the miRNA and that of its target has been confirmed experimentally.

Supplementary Tables

Suppl. Table S1 The nucleotide data used for transcriptome assembly.

Suppl. Table S2 Statistics of the Unigene.

Suppl. Table S3 Statistics of sequencing reads from the four *A. mongolicus* small RNA libraries.

Suppl. Table S8 Putative homologs of the non-conserved *A. mongolicus* miRNAs identified from eight legume species. The sequence of miRNA mature form is in upper case. The different nucleotides between *A. mongolicus* miRNA and its predicted homologs are highlighted in green.

Suppl. Table S12 Primers used for SL-qRT-PCR analyses.

Suppl. Table S13 Primers used for qRT-PCR analyses of miRNA targets.

Suppl. Table S1 The nucleotide data used for transcriptome assembly.

Sequencing technologies	Accession numbers	Databases	Raw sequence quantity	Total clean nucleotides (nt)
Sanger sequencing	-	NCBI EST	5801	4.7 M
454 pyrosequencing	SRR486144	NCBI SRA	672,002	374.1 M
454 pyrosequencing	SRR901768	NCBI SRA	522,838	139.6 M
454 pyrosequencing	SRR900017	NCBI SRA	544,678	136.0 M
Illumina	SRR647044	NCBI SRA	132,362,776	11.1 G
Illumina	SRR1035932	NCBI SRA	68,482,084	5.3 G

Suppl. Table S2 Statistics of the Unigene.

Parameters	Number
Number of contig	129,558
Number of large contigs (≥1000bp)	35,506
Max contig length (bp)	14,806
Mean contig length (bp)	827
N50 (bp)	1,400
Total bases (MB)	107.1

Suppl. Table S3 Statistics of sequencing reads from the four *A. mongolicus* small RNA libraries.

Libraries	CK_R				CK_L				DT_R				DT_L			
	Number of total sequences	% of total sequences	Number of unique sequences	% of unique sequences	Number of total sequences	% of total sequences	Number of unique sequences	% of unique sequences	Number of total sequences	% of total sequences	Number of unique sequences	% of unique sequences	Number of total sequences	% of total sequences	Number of unique sequences	% of unique sequences
Raw reads	12,084,271	100.00	2,722,952	100	11,050,444	100.00	3,300,786	100.00	12,954,184	100.00	2,356,951	100.00	11,823,128	100.00	1,547,682	100.00
3′ adaptor & length filter	2,831,243	23.43	895,498	32.89	1,039,039	9.40	475,102	14.39	8,074,144	62.33	1,578,240	66.96	6,558,179	55.47	1,058,902	68.42
Junk reads	19,677	0.16	15,604	0.57	39,009	0.35	32,006	0.97	8,499	0.07	5,530	0.23	5,749	0.05	4,246	0.27
Rfam	715,229	5.92	133,743	4.91	420,449	3.80	56,466	1.71	832,514	6.43	110,424	4.69	347,770	2.94	37,326	2.41
Repeats	12,417	0.10	3,336	0.12	7,432	0.07	1,564	0.05	15,621	0.12	3,354	0.14	13,695	0.12	2,186	0.14
Clean reads	8,508,805	70.41	1,675,514	61.53	9,545,909	86.38	2,735,952	82.89	4,026,577	31.08	660,161	28.01	4,899,378	41.44	445,405	28.78
rRNA	447,587	3.70	80,270	0.66	328,372	2.97	40,841	0.37	393,995	3.04	58,964	0.46	252,909	2.14	24,343	0.21
tRNA	216,135	1.79	40,100	0.33	67,541	0.61	8,955	0.08	387,022	2.99	39,846	0.31	66,640	0.56	6,808	0.06
snoRNA	9,275	0.08	2,580	0.02	3,363	0.03	1,339	0.01	11,264	0.09	2,366	0.02	6,488	0.05	1,408	0.01
snRNA	8,887	0.07	3,281	0.03	2,400	0.02	1,328	0.01	7,432	0.06	2,645	0.02	2,554	0.02	1,218	0.01
other Rfam RNA	33,345	0.28	7,512	0.06	18,773	0.17	4,003	0.04	32,801	0.25	6,603	0.05	19,179	0.16	3,549	0.03

Suppl. Table S8 Putative homologs of the non-conserved *A. mongolicus* miRNAs identified from eight legume species. The sequence of miRNA mature form is in upper case. The different nucleotides between *A. mongolicus* miRNA and its predicted homologs are highlighted in green.

miRNA	Stem-loop sequence of the non-conserved *A. mongolicus* miRNA	Organism	Stem-loop sequence of the putative miRNA homolog identified from eight legume species
miR-P38, miR-P39	uuaugcagcaaaacucccccuccaccauuacuuuaaucAAAUGUCAGCCGUUGGAUUAAucuaacccauggagaaagauuucucagcuguuagauuAAUCCAACGGCUGACAUUUGAcuagugggaguuuugccacagag	*Medicago truncatula*	auggcuugguccaaccAAAUGUCGGUAGUUGGAUUGUucuaacgguugagaguauaucucucaaccguuaaaauAAUCCAACGGCAGACAUUUGAauggauugaacuag
miR-P40, miR-P41	aauagauaacUCAUUAAUCAUCUACUGAACUauauuaaccauccacugauauacucauuaaugguuaauacagUUCAGUGGAUGGUUAGUGAGUuacccauuua	*Cicer arietinum*	auauugcaacUCAUUAAUCAUCUACUGAACAuuauuaacuacaaauuuaaauaugaucaacuuuaauuuuuagugucaaaucuacauggguugaaacacuucaaauugaaguuaauaaauauuuaaauuuguaauuaauuaaaUUCAGUUGAUGAUUAAUGAGUuaucuaau
		Medicago truncatula	auuugguugguuaauacaguucaaaaucauugucaauuaugUUCAAUUGAUGAUUAAUGAGUugcgacauguuguuagauUCAUUAAUCAUCUACUGAACuuaauuaaccucauauuuaaaccgcguuaaccacccaaauu
miR-P49, miR-P50	caaauacgugGUUAAUUACGUUCAGUGGAUGauuaaugaguugugauccuuuauuacauccauuaaccaUCCACUGAACGUAAUUAACCAcguauuugaa	*Cicer arietinum*	caaauguguaGUUAAUUACGUUCAGUGGAUGguuaaugaauuguaacacuuuguuauauucacuaaccaUCCAAUGAAUGUAAUUAACCAc
		Medicago truncatula	caaaauugugGUUAAUUACGUUCAGUGGAUGauuaaugaguugugacacuuuguuauauucauuaaccaUCCACUUAACGUAACUAACCAuauauucaaag
		Medicago truncatula	caaaaucgugGUUAAUUAAGUUCAUCUGCAGAUGguugaugagcugugacaccuuauuauauucauuauccaUCCACUGAACGUAAUUAACCAcguauucaaa
miR-P61,miR-P62	uaagggucUGUUUGGGAGAAGUGGGAAAGaggggaaaucuuacagaaaguacuaugcuuucccucccuccucuuacUUUUCCGCCUCUUUCAAACAGacccgcagu	*Lotus japonicus*	uaagggucUGUUUGAGAGAAGUGGGAAAGagggggaaagacuacaaaauguaguaugcuuucucccccuuucucaCUUUCCCACUUCUUUCAAACAGgccc
miR-C5, miR-C6	ucucucuuaaUGAUUGAAAUUCAUGUGGGUCcuacuaaauuuaagagggacccauaugaauuagugggaCUCACAUGAAUUUCAACCAAUGgaaagagu	*Cicer arietinum*	acuccuucauauUGGUUAAAAUUCAUGUGGGACccaccaaauuaugugggucccauuuaaauuuagcgagaCUCACAUGAAUUUCAACCAAUAugaga
		Lotus japonicus	cuuuuauugauUGGUUAAAAUUCAUGUGAGUCuacuaaauauaagagucucacuuccaauuuagugagaCUCACAUGAAUUUCAACCAAU
miR-C46	aaauuacaugguuaacgcaguucaaauauauaAUUAAUUACGUUCAGUGGAUGacuaaugaguugugaccccuuauuauauccauuaaccauccacugaauguaauuaaccacguauuugaacugcguuaaccauguaaagc	*Cicer arietinum*	augguuaacacaguucaaaugugu aGUUAAUUACGUUCAGUGGAUGguuaaugaauuguaacacuuuguuauauucacuaaccauccaaugaauguaauuaaccac
		Medicago truncatula	caugguuaaugaaguucaaaauugugGUUAAUUACGUUCAGUGGAUGauuaaugaguugugacacuuuguuauauucauuaaccauccacu

			uaacguaacuaaccauauauu
miR-C49	auacuagcaacacgcucucuaacacucuuuuuccaacaaacucuuuccuauuga uugaaauucaugugggguccuacaaaauucaaguggga cccauauaaauuUUA ACCAAUAGAAGAGAGUGUguugaaaauagaguguuaaagagugc guugcuagcac	*Cicer arietinum*	auacuagcaacacacucuuugacacacauuuuagcacacucucuuuuauugg uuaaaauucacaugggguccuaacaaaauuagaugggacccacaauuuggugg gacucauguaaauuUCAACCAAUAGAAGAGAGUGUguug
		Medicago truncatula	augcuagcaacacacauucuaacacacuuuccaacacacucucuucaauugg uuaaaaaucacaugggacccaccaaauuuauaugggacccacaugauuuggu gggacccauaugaauuUUAACCAAUCGAAGAGAGUGUguu ggaaagugugugu uagaaugugug uugcuagca
miR-C50	aaagcucaucuaaaguaaccaagaguauaaagugagaaauuaaagauguauuca ccuuuguuacuuuaaguggauugaaaucucaauauacuuuaacuAAUCAA UGGUGGAUACAUCCUuacuuucucauuuuacauauuuguucacuuu agaggagccua	*Phaseolus vulgaris*	uacucucuagagugaguuuguaucuaaagugcacaagaguguauccacacu ugaucuuaaaaaaguauaucaugaucuuacccacucaaAUUCAAUGG UGGAUACAUCCUuauuugugcaauuuauagauaagcucauuuuag aggagccu

Suppl. Table S12 Primers used for SL-qRT-PCR analyses.

miRNA ID	miRNA sequences (5' to 3')	Reverse transcription primers (5' to 3')	Forward primers (5' to 3')	Reverse primers (5' to 3')
miR1507-1	UCUCAUUCCACACAUCGUCUAA	GTCGTATCCAGTGCAGGGTCCGAGGTATTCGCACTGGATACGACTTAGAC	CGGCTCTCATTCCACACATC	CAGTGCAGGGTCCGAGGTAT
miR1509-1	UUAAUCAAGGAAAUCACAGUCG	GTTGGCTCTGGTGCAGGGTCCGAGGTATTCGCACCAGAGCCAACCGACTG	GTGGGGTTAATCAAGGAAATCA	GTGCAGGGTCCGAGGT
miR1511-1	AACCAGGCUCUGAUACCAUGA	GTTGGCTCTGGTGCAGGGTCCGAGGTATTCGCACCAGAGCCAACTCATGG	GTGGAACCAGGCTCTGATA	GTGCAGGGTCCGAGGT
miR1515-1	UCAUUUUUGCGUGUAAUGAUCC	GTTGGCTCTGGTGCAGGGTCCGAGGTATTCGCACCAGAGCCAACGGATCA	GTTGGGTCATTTTTGCGTGTAA	GTGCAGGGTCCGAGGT
miR156-1	UUGACAGAAGAUAGAGAGCAC	GTCGTATCCAGTGCAGGGTCCGAGGTATTCGCACTGGATACGACGTGCTC	CCGCGTTGACAGAAGATAGAGA	CAGTGCAGGGTCCGAGGTA
miR159-1	UUUGGAUUGAAGGGAGCUCU	GTTGGCTCTGGTGCAGGGTCCGAGGTATTCGCACCAGAGCCAACAGAGCT	GTGGGTTTGGATTGAAGGG	GTGCAGGGTCCGAGGT
miR159-3	UUUGGAUUGAAGGGAGCUCAA	GTCGTATCCAGTGCAGGGTCCGAGGTATTCGCACTGGATACGACTTGAGC	CGGCGGTTTGGATTGAAG	CAGTGCAGGGTCCGAGGTAT
miR164-1	UGGAGAAGCAGGGCACGUGCA	GTTGGCTCTGGTGCAGGGTCCGAGGTATTCGCACCAGAGCCAACTGCACG	GTTTTGGAGAAGCAGGGCA	GTGCAGGGTCCGAGGT
miR166-1	UCGGACCAGGCUUCAUUCCCC	GTCGTATCCAGTGCAGGGTCCGAGGTATTCGCACTGGATACGACGGGGAA	CGGTCGGACCAGGCTTCATT	CCAGTGCAGGGTCCGAGGTATTC
miR167-1	UGAAGCUGCCAGCAUGAUCUGA	GCGTGGTCCACACCACCTGAGCCGCCACGACCACGCTCAGATCA	GAGCCGTGAAGCTGCCAG	TCCACACCACCTGAGCCG
miR168-1	UCGCUUGGUGCAGGUCGGGAA	CTCAGCGGCTGTCGTGGACTGGGTGCTGCCGCTGAGTTCCCGACTTCCCG	CGGTGTGTCGCTTGGTGC	GGCTGTCGTGGACTGGGTG
miR169-1	CAGCCAAGAAUGACUUGCCGG	GTTGGCTCTGGTGCAGGGTCCGAGGTATTCGCACCAGAGCCAACCCGGCA	GTGGCAGCCAAGAATGACT	GTGCAGGGTCCGAGGT
miR171-1	UGAUUGAGCCGUGCCAAUAUC	GTTGGCTCTGGTGCAGGGTCCGAGGTATTCGCACCAGAGCCAACGATATT	GTTTTGATTGAGCCGTGCC	GTGCAGGGTCCGAGGT
miR2111-1	UAAUCUGCAUCCUGAGGUUU	GTTGGCTCTGGTGCAGGGTCCGAGGTATTCGCACCAGAGCCAACAAACCT	GTGGGTAATCTGCATCCTG	GTGCAGGGTCCGAGGT
miR2118-1	UUUCCGAUUCCACCCAUUCCUA	GTCGTATCCAGTGCAGGGTCCGAGGTATTCGCACTGGATACGACTAGGAA	AAGGCGTTTCCGATTCCA	CAGTGCAGGGTCCGAGGTAT
miR319-1	UUGGACUGAAGGGAGCUCCC	GTTGGCTCTGGTGCAGGGTCCGAGGTATTCGCACCAGAGCCAACGGGAGC	GTTGGTTGGACTGAAGGGA	GTGCAGGGTCCGAGGT
miR390-1	AAGCUCAGGAGGGAUAGCGCC	GTTGGCTCTGGTGCAGGGTCCGAGGTATTCGCACCAGAGCCAACGGCGCT	GTTGAAGCTCAGGAGGGAT	GTGCAGGGTCCGAGGT
miR393-1	UUCCAAAGGGAUCGCAUUGAUU	GTTGGCTCTGGTGCAGGGTCCGAGGTATTCGCACCAGAGCCAACAATCAA	GTGTTCCAAAGGGATCGCA	GTGCAGGGTCCGAGGT

miR394-1	UUGGCAUUCUGUCCACC UCC	GTTGGCTCTGGTGCAGGGTCCGAGGTATTCGCACCAGAGC CAACGGAGGT	GTTGGTTGGCATTCTGTC C	GTGCAGGGTCCGAGGT
miR395-3	UGAAGUGUUUGGGGGAA CUCC	GTTGGCTCTGGTGCAGGGTCCGAGGTATTCGCACCAGAGC CAACGGAGTT	GTTGTGAAGTGTTTGGGG G	GTGCAGGGTCCGAGGT
miR396-1	UUCCACAGCUUUCUUGA ACUG	GTTGGCTCTGGTGCAGGGTCCGAGGTATTCGCACCAGAGC CAACCAGTTC	GGGGTTCCACAGCTTTCT T	GTGCAGGGTCCGAGGT
miR397-1	AUCGACGCUGCACUCAA UCAU	GTTGGCTCTGGTGCAGGGTCCGAGGTATTCGCACCAGAGC CAACATGATT	GTTTATCGACGCTGCACT C	GTGCAGGGTCCGAGGT
miR398-1	UGUGUUCUCAGGUCGCC CCUG	GTCGTATCCAGTGCAGGGTCCGAGGTATTCGCACTGGATAC GACCAGGGG	CGGCGGTGTGTTCTCAGG T	CAGTGCAGGGTCCGAGGT AT
miR398-2	UUGUGUUCUCAGGUCAC CCCU	GTTGGCTCTGGTGCAGGGTCCGAGGTATTCGCACCAGAGC CAACAGGGGT	GTGGTTGTGTTCTCAGGT C	GTGCAGGGTCCGAGGT
miR403-1	UUAGAUUCACGCACAAA CUUG	GTTGGCTCTGGTGCAGGGTCCGAGGTATTCGCACCAGAGC CAACCAAGTT	GGGGTTAGATTCACGCAC A	GTGCAGGGTCCGAGGT
miR408-1	AUGCACUGCCUCUUCCCU GGC	GCGTGGTCCACACCACCTGAGCCGCCACGACCACGCGCCA GGGA	CCAGCAATGCACTGCCTC TT	TCCACACCACCTGAGCCG
miR482-1	UUCCCAAAGCCGCCCAUU CCGA	GTTGGCTCTGGTGCAGGGTCCGAGGTATTCGCACCAGAGC CAACTCGGAA	TTGTTCCCAAAGCCGCCC	GTGCAGGGTCCGAGGT
miR5037-1	AACCCUCAGAGGCUUCCA CGG	GTTGGCTCTGGTGCAGGGTCCGAGGTATTCGCACCAGAGC CAACCCGTGG	GTTGAACCCTCAGAGGC TT	GTGCAGGGTCCGAGGT
miR5225-1	CCUGUCGCAGGAGAGAU GACG	GTTGGCTCTGGTGCAGGGTCCGAGGTATTCGCACCAGAGC CAACCGTCAT	TTGCCTGTCGCAGGAGA G	GTGCAGGGTCCGAGGT
miR858-1	CUCGUUGUCUGUUCGAC CUUG	GTTGGCTCTGGTGCAGGGTCCGAGGTATTCGCACCAGAGC CAACCAAGGT	GTTGCTCGTTGTCTGTTC G	GTGCAGGGTCCGAGGT
U6	-	AGAAGGCACAAAAGAAAAGGC	TTATTGATTCAGCAGTGG AGGTT	AGAAGGCACAAAAGAAA AGGC

Suppl. Table S13 Primers used for qRT-PCR analyses of miRNA targets.

Target transcript ID	miRNA	Transcript Annotation	Forward primer (5' to 3')	Reverse primer (5' to 3')
comp2829_c0_seq1	miR2118-1	*Zinc finger CCCH domain-containing protein 20-like* [*Glycine max*]	GGATCTCGGAACTGGTGAAG	CAGACCAAGCTGTCTCATGC
comp25810_c0_seq1	miR-P11	*RNA-binding protein 42*	CAAGGGAGGAAGAAAGCTGA	CAGGGGCCTCAATTTCTCTA
comp108786_c0_seq1	miR-P14	*Ethylene-responsive transcription factor-like protein At4g13040*	TCTTGGCAGTGACACGACAT	TGCTCTGCATTTTCACAAGC
comp68654_c0_seq1	miR858-1	*Transcription factor MYB12-like* [*Glycine max*]	GGGCCATGGTGTATCTTCTG	TTCCATTGAGTCCCCGTTAC
comp68636_c0_seq1	miR858-1	*Transcription factor MYB12-like* [*Glycine max*]	GACAGTAGTTGCGTGCTGGA	TTGTGAGGGCAAGCATAGTG
comp68647_c0_seq1	miR858-1	*Transcription factor MYB12*	GGGCCATGAAGAAGAACAAA	CCGTCAGGGTTCAAAATCAT
comp6528_c0_seq1	miR858-1	*MYB-related protein MYB4*	CAATGAGACAAGCAGCGATT	TCGCATAAACGTCGTACCAA
comp10661_c0_seq1	miR159 and miR319	*Transcription factor GAMYB-like* [*Glycine max*]	CAACATGCCTGCTGTCTGTC	AAATCTTTCTCAGCCCAGGAG
comp23575_c0_seq1	miR159 and miR319	*Transcription factor GAMYB-like* [*Glycine max*]	GATATTGCTTTGGCCTCTGG	CATGACTGAAGGCTCTGAAGG
comp10257_c0_seq1	miR156	*Squamosa promoter-binding-like protein 12-like* [*Glycine max*]	GTGTGCATGCTGTTTGGCTA	TGACAGCAAGGTAGACAAGGAA
comp1884_c0_seq1	miR164	*NAC domain-containing protein* [*Ammopiptanthus mongolicus*]	GGTATGCCCCACTTGTGGAA	TTGGTGGCATGAGGGAAGAG
comp45112_c0_seq1	miR167	*Auxin response factor 8-like* [*Glycine max*]	TCCTAGTTGAGTGGCCGAAC	ACCAAATTACAAGAATGCCGCA
comp124400_c0_seq1	miR169	*Nuclear transcription factor Y subunit A-1-like* [*Glycine max*]	CAAGCCTGAGATGGATGGTCA	ACAACCGCTGAGTTACTACACA
comp2685_c0_seq1	miR396	*Growth-regulating factor 9-like isoform X2* [*Glycine max*]	TAAGAAGTGGCGGTGCAAGA	AGCTGGTTCATGTTCTGCGA
comp51601_c0_seq1	miR396	*Cysteine proteinase RD21a-like* [*Glycine max*]	ACAGTGCAGTTGGAGGTGAG	TGGACTAGTGTTGCAGTGCT
comp37827_c0_seq1	miR408	*Plantacyanin*	CGGTGAACAAGGCTGGGTAT	TCCCCTAGCAAGCCTGATCT
-	-	*AmeIF1*	CTGACATGCGCCGTAGGAACG	CCCTGCTTATGCCAGTCTTTT
